# The amyloid interactome: Exploring protein aggregation

**DOI:** 10.1371/journal.pone.0173163

**Published:** 2017-03-01

**Authors:** Konstantina V. Biza, Katerina C. Nastou, Paraskevi L. Tsiolaki, Chara V. Mastrokalou, Stavros J. Hamodrakas, Vassiliki A. Iconomidou

**Affiliations:** Section of Cell Biology and Biophysics, Department of Biology, School of Sciences, National and Kapodistrian University of Athens, Panepistimiopolis, Athens, Greece; Deutsches Zentrum fur Neurodegenerative Erkrankungen, GERMANY

## Abstract

Protein-protein interactions are the quintessence of physiological activities, but also participate in pathological conditions. Amyloid formation, an abnormal protein-protein interaction process, is a widespread phenomenon in divergent proteins and peptides, resulting in a variety of aggregation disorders. The complexity of the mechanisms underlying amyloid formation/amyloidogenicity is a matter of great scientific interest, since their revelation will provide important insight on principles governing protein misfolding, self-assembly and aggregation. The implication of more than one protein in the progression of different aggregation disorders, together with the cited synergistic occurrence between amyloidogenic proteins, highlights the necessity for a more universal approach, during the study of these proteins. In an attempt to address this pivotal need we constructed and analyzed the human amyloid interactome, a protein-protein interaction network of amyloidogenic proteins and their experimentally verified interactors. This network assembled known interconnections between well-characterized amyloidogenic proteins and proteins related to amyloid fibril formation. The consecutive extended computational analysis revealed significant topological characteristics and unraveled the functional roles of all constituent elements. This study introduces a detailed protein map of amyloidogenicity that will aid immensely towards separate intervention strategies, specifically targeting sub-networks of significant nodes, in an attempt to design possible novel therapeutics for aggregation disorders.

## Introduction

The cellular and molecular mechanisms that underlie protein misfolding are a matter of major concern for studies conducted in several scientific centers all over the world. Under denaturing conditions, a growing number of proteins and peptides that fail to fold properly into their native structure, are led to the formation of highly ordered, insoluble aggregates, the so-called amyloid fibrils [1]. Amyloidogenicity, the ability of proteins to self-assemble into these well-defined fibrillar structures, was initially associated with a group of functionally unrelated proteins [2]. Meanwhile, targeted *in vitro* experiments revealed that amyloid formation is a universal phenomenon for polypeptide chains [3] and thus, this concept was the onset of a new era in protein misfolding, since a great number of novel amyloidogenic proteins and peptides were uncovered [4]. Noteworthy, proteins, ranging from bacteria to humans, have been also found to adopt the same amyloid architecture, as part of their nature [5, 6]. A vast amount of data, regarding amyloid fibril formation, present both in pathological and physiological conditions, is currently organized into freely available databases [7–11].

Amyloid fibril formation is widely observed and directly linked to the pathology of a range of widespread human diseases, known as amyloidoses [2]. Amyloidoses are a group of aggregation-disorders, where full-length amyloidogenic proteins or fragments of larger amyloidogenic protein precursors, precipitate and deposit, forming amyloid plaques and resulting in organ or tissue dysfunction [12, 13]. Literature data indicate the implication of more than one amyloidogenic proteins in the evolution of different amyloidoses. In the case of Senile Systemic Amyloidosis, co-operation of several Apolipoproteins and ATTR is recorded [14, 15], whereas in Alzheimer’s disease, apart from Aβ, proteinaceous components such as ACys, ATTR and AGel were found [16]. To date, the extent to which co-deposition in amyloid plaques has impacted the development of amyloidoses between putative unrelated amyloidogenic proteins, remains unclear.

Experimental work over the past ten years has revealed an intriguing, synergistic phenomenon between amyloidogenic proteins [17]. *In vitro* experiments highlighted the capacity of Aβ peptide under specific conditions to seed the polymerization process for α-synuclein [18], Tau [19] or APrp protein [20]. Similar experiments were performed on several well-characterized amyloidogenic proteins [21–23]. Further to *in vitro* assays, animal models demonstrated the co-deposition of Aβ and Tau proteins [24] or APrp protein [20] in transgenic models. However, a hidden perspective emerges from this molecular association; amyloid “cross-seeding” could explain mechanistically the way by which misfolded proteins co-deposite, and propose possible, attractive candidates for the development of novel therapeutic strategies of aggregation-related diseases. An apt example towards this direction is the protective role of the amyloidogenic ACys in neurodegenerative diseases [25].

The interactomes [26, 27], a systems biology approach, were viable complements to proteomics, in an attempt to look at “the big picture” of protein-protein interactions (PPIs). Gaining a proper understanding of PPIs contributed to several problems in the field of biological and medical research [28–30] and served as a reference for further targeted experimentation [31]. Systematic PPI studies are essential, in order to fully comprehend the molecular mechanisms that trigger human diseases [32, 33]. However, a subject poorly explored so far is deviating PPIs associated with amyloidogenic/amyloid forming proteins. To date, only a few studies utilized a protein interaction network framework, to obtain information regarding the Alzheimer’s [34–36] or Huntington’s disease [37] and to construct the Amyloid precursor protein interactome [38–40].

Incomplete knowledge on direct and/or indirect interactions of proteins “prone-to-misfold”, emphasizes the need to focus on the amyloid protein-protein interaction network. Here we introduce the amyloid interactome, a systematic approach to study “macroscopically” interactions between previously unrelated human amyloidogenic proteins, associated with distinct pathologies. Our ultimate goal was to find a common denominator for amyloid formation, unveil the relationships that govern amyloidogenicity and, subsequently, guide further experimental studies on protein misfolding.

## Materials and methods

### Amyloid classification

In order to classify amyloidogenic proteins, all protein-precursors were sorted into three categories:

- *in vivo* amyloid forming protein: the precursor protein, or a peptide segment–derived from the precursor protein–, self assembles into typical amyloid fibrils, affecting one or more tissues or organs in human. These proteins, from a clinical perspective, give rise to distinct amyloidoses or play a pathological role in neurodegenerative or endocrine diseases [2].
- *in vitro* amyloid forming protein: the precursor protein, or commonly a peptide segment–derived from the precursor protein–, that was reported to self assemble into amyloid-like fibrils, at experimental level. The amyloidogenicity of proteins comprising this list may be speculative. Only human precursor proteins are mentioned in this category.
- protein related to amyloid fibril formation: the protein is associated with other *in vivo* amyloid forming proteins, but has no amyloid properties recorded.

### Amyloid interactome datasets

Amyloidogenic proteins were firstly obtained from a literature-curated dataset, peer-reviewed in 2014, by the International Society of Amyloidosis (http://www.amyloidosis.nl/). This list included human proteins known to self-assemble into typical amyloid fibrils *in vivo*, along with intracellular inclusions with known biochemical composition [2]. In addition to this, the set was enriched with proteins that form amyloid fibrils *in vitro* [41]. To expand this dataset, AmyLoad [7] was used as a source of supplementary proteins, characterized to form amyloid-like fibrils *in vitro* at experimental level. A final addition included several UniProtKB [42] entries, gathered elaborately to incorporate reviewed proteins related to amyloid fibrils. Overall, the dataset contained 145 non-redundant amyloidogenic protein precursors. S1 Table provides a detailed catalogue of the aforementioned proteins, mapped to a UniProtKB Accession Number (AC).

The subsequent construction of the network incorporated only well-characterized *in vivo* amyloidogenic proteins, published by Sipe et al. [2], excluding Enfurvitide an anti-retroviral peptide drug [43], as well as Immunoglobulin Light and Heavy Chains. In the case of Immunoglobulin chains (e.g. Bence Jones proteins [44]), their variety in human population did not allow the identification of a unique protein precursor, related to amyloid fibril formation. The final seed-dataset included 28 proteins, related to *in vivo* amyloid fibril formation (Table 1), which were subsequently used for the collection of protein-protein interactions. Protein nomenclature follows abbreviations established by Sipe et al. [2].

**Table 1:** The dataset of 28 proteins related to in vivo amyloid fibril formation.

Protein Precursor Name*	Abbreviation	UniProtKB AC
Amyloid beta A4 protein	Aβ	P05067
Apolipoprotein A-I	AApoAI	P02647
Apolipoprotein A-II	AApoAII	P02652
Apolipoprotein A-IV	AApoAIV	P06727
Beta-2-microglobulin	Aβ2M	P61769
Calcitonin	ACal	P01258
Corneodesmosin	ACor	Q15517
Cystatin-C	ACys	P01034
Fibrinogen alpha chain	Afib	P02671
Galectin 7	AGal	P47929
Gelsolin	AGel	P06396
Insulin	AIns	P01308
Integral membrane protein 2B	ABri/ ADan	Q9Y287
Islet Amyloid Polypeptide	AIAPP	P10997
Kerato-epithelin	Aker	Q15582
Lactadherin	AMed	Q08431
Lactoferrin	ALac	P02788
Leukocyte cell-derived chemotaxin-2	ALECT2	O14960
Lysozyme C	Alys	P61626
Major prion protein	APrP	P04156
Natriuretic peptides A	AANF	P01160
Odontogenic Ameloblast-Associated Protein	AOAAP	A1E959
Prolactin	APro	P01236
Pulmonary surfactant associated protein C	APSP	P11686
Semenogelin-1	ASem1	P04279
Serum amyloid A-1	AA1	P0DJI8
Serum amyloid A-2	AA2	P0DJI9
Transthyretin	ATTR	P02766

*Protein nomenclature follows abbreviations published by Sipe *et al*.

### Assembling the protein-protein interaction dataset

UniProtKB ACs were used to query IntAct [45], BioGRID [46] and STRING [47] databases, in order to extract experimentally verified PPIs for the 28 proteins related to *in vivo* amyloid fibril formation (Table 1). This process resulted in three independent PPI datasets, derived from each database (data not shown). In general protein-protein interaction data contain experimentally verified interactions, along with data derived from prediction methods. These last data do not have the high reliability often attributed to them and thus, in order to avoid extracting automatic text-mining results from the plethora of scientific articles related to amyloid fibril formation, BioGRID and STRING datasets were excluded from any further analysis. IntAct PPIs, gather highly curated experimental data, which ensured the quality and consistency of information of our dataset [48].

The interaction data from IntAct (05–2016) were retrieved in a MITAB 2.5 format file [49], which is appropriate for Perl parsing, without the loss of information regarding PPIs. An editing process of the file allowed the removal of all the non-human interactions, and additional screening was performed to dismiss interactions with chemical compounds. The resulting set included 355 protein nodes with 762 edges.

In order to create a more robust network, the interactions deposited in IntAct between all 355 proteins were retrieved, whilst an extra processing allowed for the removal of self-loops and duplicated edges. Thus, a final dataset of 1178 PPIs between 353 human proteins was obtained, after the exclusion of two protein nodes that had only self interactions (See Results and discussion).

### Visualization and analysis of the network

For the visualization and the analysis of the network we followed the protocol introduced by Nastou et al. [50]. Cytoscape 3.2.1 [51] was used to manipulate, analyze and visualize our data, since it ideally provides all the necessary and improved applications for the analysis of biological networks [52]. The analysis of simple and complex network topology parameters, was performed by NetworkAnalyzer [53]. The Cytoscape.js JavaScript library [54] was used to create interactive networks, is available at this link: http://83.212.109.111/amyloid_interactome.

Clustering analysis was performed with clusterMaker [55], utilizing the Markov Clustering algorithm (MCL), an optimal choice for biological interaction networks assembled from high-throughput experiments. Different inflation values, between 1.8 and 3.0, were used during the clustering process (data not shown). The inflation value was finally set to 1.8, as it has been proved to be the most suitable for biological networks [50, 56], in consistency with the observation that values above 1.8, result in extreme network fragmentation and the creation of clusters with minor biological significance (See Results and discussion).

BiNGO [57], an application for Cytoscape and WebGestalt [58], an online server, were both used to perform a functional enrichment analysis of the network. BiNGO determines Gene Ontology (GO) [59] categories that are statistically overrepresented in a set of proteins in a biological network (e.g. a cluster), and thus, aids in the detection of functional modules. WebGestalt was used to further supplement this analysis, since, besides GO term it can perform Disease Association and KEGG pathway analysis. The hypergeometric method was used, and significance was set at an adjusted P-value of <0.05 for BiNGO and <0.01 for WebGestalt (Benjamini and Hochberg method). Significant categories, driven by only two or less proteins, were discarded due to the high potential for false signals in such cases. S1 Fig outlines the overall study design of the amyloid interactome (S1 Fig).

## Results and discussion

**Fig 1:**
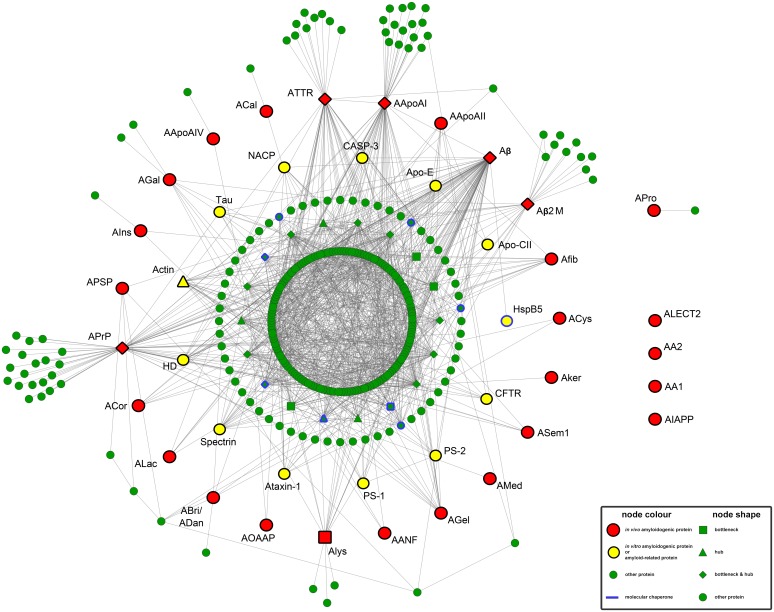
The amyloid interactome. Interaction data for the creation of this network were gathered from the publicly available database IntAct [45] and Cytoscape [51] was used as a visualization tool (Interactive network available at http://83.212.109.111/amyloid_interactome). The network consists of 353 nodes and 1178 edges. Proteins are depicted as nodes and interactions as edges. Red-coloured nodes represent known *in vivo* amyloidogenic proteins, whereas yellow-coloured nodes represent *in vitro* amyloid-forming proteins or proteins related to amyloid fibril formation (see also Tables 1 and 2). Green-coloured nodes are proteins, listed as other interaction partners. Hubs and bottlenecks are depicted as triangles (▲) and squares (■), respectively. Protein-nodes, which are both hubs and bottlenecks are shown as diamonds (◆). Important molecular chaperones are highlighted with a blue outline.

The study of the dynamics, structure and function of protein-protein interaction networks (PPINs) has proven crucial for the understanding of many biological phenomena [60–63]. Hence, network theory is a sophisticated approach to study the puzzling phenomenon of amyloidogenicity. The amyloid interactome displays the interacting partners of *in vivo* amyloid forming proteins in a flat and detailed protein map (Fig 1). The results presented in this work, combine interactions from specialized networks of protein aggregation [34, 35, 37–40, 64] and eventually, assemble a new set of functionally unconnected proteins into a network that would possibly fill the missing pieces of protein aggregation and shed light towards the exploitation of novel disease protein-targets.

### Construction of the amyloid interactome

In an effort to build the human amyloid interactome, *in vivo* amyloid-forming proteins were obtained from a peer-review library, published by Sipe et al. [2] (Table 1). Overall the protein-protein interaction network contains 353 protein nodes and 1178 protein-protein interaction edges, between them (Fig 1). Surprisingly, among the proteins listed in the network, 23 amyloidogenic proteins construct a giant connected component (Fig 1, red nodes), whereas 13 proteins (Fig 1, yellow nodes), recorded as *in vitro* amyloid-forming proteins and proteins related to amyloid fibril formation (S1 Table), are also identified. A list of these significant proteins is available in Table 2. Among them, proteins forming intracellular inclusions bodies were reported (Tau, Actin, NACP, HD) [2], while proteins, found as co-deposits in the Alzheimer’s disease, emerged (Apo-E, PS-1, PS-2, Tau) [65].

**Table 2:** The dataset of 13 proteins related to amyloid fibril formation.

Protein Precursor Name*	Abbreviation	UniProt AC
***In vitro amyloid-forming protein precursors***		
alpha beta Crystallin (ABC)	HspB5	P02511
alpha-Synuclein	NACP	P37840
Apolipoprotein C-II	Apo-CII	P02655
Caspase-3 precursor	CASP-3	P42574
Cystic fibrosis transmembrane conductance regulator	CFTR	P13569
Huntingtin (Polyq expanded)	HD	P42858
Presenilin 1	PS-1	P49768
Presenilin 2	PS-2	P49810
Spectrin SH3	Spectrin	Q13813
Tau	Tau	P10636
***Proteins related to amyloid fibril formation***		
Actin, cytoplasmic 1	Actin	P60709
Apolipoprotein E	Apo-E	P02649
Ataxin 1	Ataxin-1	P54253

*Protein nomenclature follows the most cited abbreviations in literature.

Notably, APro, an anterior pituitary hormone known to self-assemble into amyloid fibrils [66], did not manage to join the giant connected component created by the other 23 amyloidogenic proteins in the network, whilst the only interactor identified was the prolactin receptor (Fig 1, right). This “detachment” of an important hormone together with the absence of AIAPP should receive a renewed emphasis [67]. Namely, for AIAPP no experimental verified partners were recorded, since only speculative approaches attempted to describe possible interaction partners [68]. AA1 and AA2 proteins, which can form amyloid fibrils after partial proteolysis [69], are important components of the High Density Lipoprotein (HDL) complex [70]. However, direct interactions of AA1 and AA2 are not recorded in IntAct. Therefore, at first glance, the network which has emerged designates a correlation among the majority of otherwise unrelated *in vivo* and also *in vitro* amyloidogenic proteins, along with proteins related to amyloid fibril formation.

### Network analysis based on graph theory

One of the most fruitful approaches to extract relative biological conclusions from the structure of the amyloid interactome is to computationally calculate its topological parameters.

#### Simple topology parameters

An assessment of simple topology parameters revealed intriguing information. A random graph with the same number of nodes and edges has been used as a “null model” to draw conclusions regarding certain topological traits of our network. In general, small-world graphs describe networks, where nodes can be reached from each other by traversing a small number of edges, and, thus their average path length is small and their clustering coefficient (transitivity) is high, compared to a random graph [71]. For the amyloid interactome, both the clustering coefficient (CC = 0.187) and the characteristic path length (CPL = 3.083) are higher and lower, respectively, than those of the corresponding random graph (CC = 0.003, CPL = 3.290), signifying that a small number of steps is needed, for one amyloidogenic protein to reach another. For example, Aβ can “reach” ATTR, by crossing AApoAI in only two (2) steps, verifying experimental data which pinpoint ATTR as a promising biomarker of the Alzheimer’s disease [72] (Fig 1, top). Furthermore, measuring the density of our network (0.019), a value lower than 0.1 was recorded, a result in accordance with other sparsely connected biological networks [73]. Since our network complies with all these criteria, we can safely conclude that it has small-world properties [74].

#### Complex topology parameters

In addition to having small-world features, biological networks are commonly scale-free [75]. The most important parameter to gain an insight on the scale-free nature of a biological network is the node degree distribution [76]. In our case, the distribution is of the following form:

$$P\left(k\right)=117.86k^{-1.236}$$ (1)

decaying as a power law (P(k) ~k^-γ^). According to this finding, the network has scale-free properties [76] and specifically it consists of a few hubs (Fig 1, triangles and diamonds) connected with multiple nodes (S2A Fig). More importantly, hubs (S2 Table) seem to play a crucial role in our network, since the degree exponent (γ) is lower than 2 [77]. Generally, networks enriched with hubs, are robust against random node deletions [78, 79] as these disturbances do not affect the average path length severely (S2D Fig). Nevertheless, the removal of particular hubs, such as the Aβ or APrP (Fig 1), can drastically alter the average path length and so, our network can be generally addressed as ‘robust yet fragile’ [80].

As shown in Fig 1, several proteins act as “bridges”, immediately connecting many, otherwise distantly or unconnected proteins in the network, thus, increasing the network’s interconnectivity (Fig 1, squares and diamonds). The influence of these “bridging” proteins is expressed with high betweeness centrality values, indicating their role as bottlenecks, key connectors for the communication of other important proteins in the interactome, like hubs [81, 82] (S2B Fig). S3 Table introduces the top 20 bottlenecks of the interactome.

Among the topological parameters mentioned above, node degree, and betweenness centrality distributions were carefully studied. Fifteen protein-nodes were identified as both hubs and bottlenecks, whereas 5 proteins have high degree (hubs) and 5 proteins have high betweenness centrality (bottlenecks). Impressively, 6 out of the 23 *in vivo* amyloidogenic proteins have a major contribution on the interactome, demonstrating their crucial role in the network (S2 and S3 Tables). Additionally, the amyloid interactome has an average clustering coefficient distribution, that follows approximately the scaling law *C(k) ~ k*^*-1*^ (S2C Fig), indicating the ability of this network to form functional modules (clusters) with biological significance (See Clustering Analysis) [83]. Overall, the network analysis based on graph theory revealed that the amyloid interactome appears to be enriched with interactions between amyloidogenic proteins.

Finally, in order to further examine the role of selected and random perturbations in the stability of the amyloid interactome, we performed a “lethality” test [78]. A multistep procedure included the gradual removal of proteins, randomly (“failure”) and in descending order of node degree and betweeness centrality (“attacks”). The rapid increase in the network’s characteristic path length (CPL) during the targeted “attacks”, in contrast to the slow increase during its “failure”, puts emphasis on the significance of the removed proteins (S2D Fig).

The above analysis gave us valuable information regarding central components of the network (hubs and bottlenecks), ranging from single proteins to entire modules (S2 and S3 Tables). However, due to inevitable technical biases present in interaction data [84], all results produced from such analyses should be carefully examined. Aβ publication biases, for example, may lead to the overestimation of the role of certain constituents of the network, in expense of others. Graph theory based analysis combined with other validation approaches were utilized, in our case, to further address the aforementioned issues.

### The amyloid interactome unravels interconnections between amyloidogenic proteins

In general, it is believed that disease-related proteins in a protein-protein interaction network are more interconnected than non-disease proteins [85], a claim in accordance with our findings. Fig 1 deciphers the complex interactions governing amyloidogenicity, by interconnecting well-characterized amyloidogenic proteins (red nodes) with a heterogeneous collection of proteins related to protein aggregation (yellow nodes). Nevertheless, it is possible to understand that not all proteins on the interactome were directly related, meaning that indirect links may occur. Consequently, amyloid forming proteins, such us Tau, NACP and HD, which were excluded from our initial seed-dataset (See Materials and methods), were ultimately retrieved during the interactome construction process (S3C Fig).

Impressively, the interaction network consolidates a number of human proteins, which have been shown to form amyloids *in vitro*. Frequently, *in vitro* aggregation assays are oriented towards protein segments, responsible to drive proteins from their native structure to the amyloid state, in place of full-length proteins [86]. Evidence at experimental level prove that “aggregation-prone” segments are indeed sufficient to lure a protein precursor into forming typical amyloid fibrils, and thus, these full-length protein precursors are characterized as “amyloidogenic” [87]. Interconnections between *in vivo* (red nodes) and *in vitro* amyloid forming proteins or protein segments (HspB5, Apo-CII, PS-1, PS-2, Spectrin, See Table 2) may extend biological expectations, related to protein-aggregation (S3A Fig).

Looking deeper into the crowded topography of the amyloid interactome, a great variety of “amyloid-binding proteins” is included. These amyloid specific molecules are basically a list of divergent proteins, capable of interacting with assemblies, derived from amyloidogenic proteins [88–90]. A broad range of Aβ contributors, for example, includes molecular chaperones or co-chaperones, apolipoproteins and other amyloid-forming proteins, enhanced with various functional characteristics. Since cells have adapted a mechanism to avoid the accumulation of incorrectly folded proteins, the Gene Ontology (GO) term [59] enrichment analysis in the entire interactome revealed the overrepresentation of GO terms regarding regulatory mechanisms (positive or negative regulation), while the most important GO term recorded is “response to stress” (GO ID: 6950) (S4 Table and S4A Fig). In particular, knowledge of the biological role of non-amyloidogenic hubs and bottlenecks in our interactome (S3B Fig), is gathered and shown in S5 Table. As expected, proteins highly interconnected are involved in signal transduction and in several metabolic processes. Impressively, though, a vast amount of topologically important proteins is related with stress pathways, highlighting possible novel disease protein targets, mediating amyloidogenicity (See Rational Design of Protein Inhibitors).

The most abundant interaction detected is the one between amyloidogenic proteins and well-known regulatory proteins, the so-called chaperones (Fig 1). As Fig 1 illustrates, chaperones together with co-chaperones dynamically participate in the interactome (nodes with blue border). This finding was, more or less, an expected phenomenon, since molecular chaperones are molecules dedicated to suppress amyloid formation [91, 92] and usually have many interactors [93]. The expert review, by Yerbury & Kumita, presents an extended group of amyloid-specific chaperones and discusses their implications [94]. Our interaction network, apart from validating existing data, demonstrates that chaperones exhibit high connectivity and at the same time high betweeness centrality, meaning that a sudden removal of such a node would result to the elimination of many important interactions in the network (Fig 1 and S2D Fig). These findings are in accordance with previously published interaction networks, associated with aging [95] or stress [96], where chaperones participate as special constituents.

### Clustering and functional enrichment analysis

#### Clustering analysis

**Fig 2:**
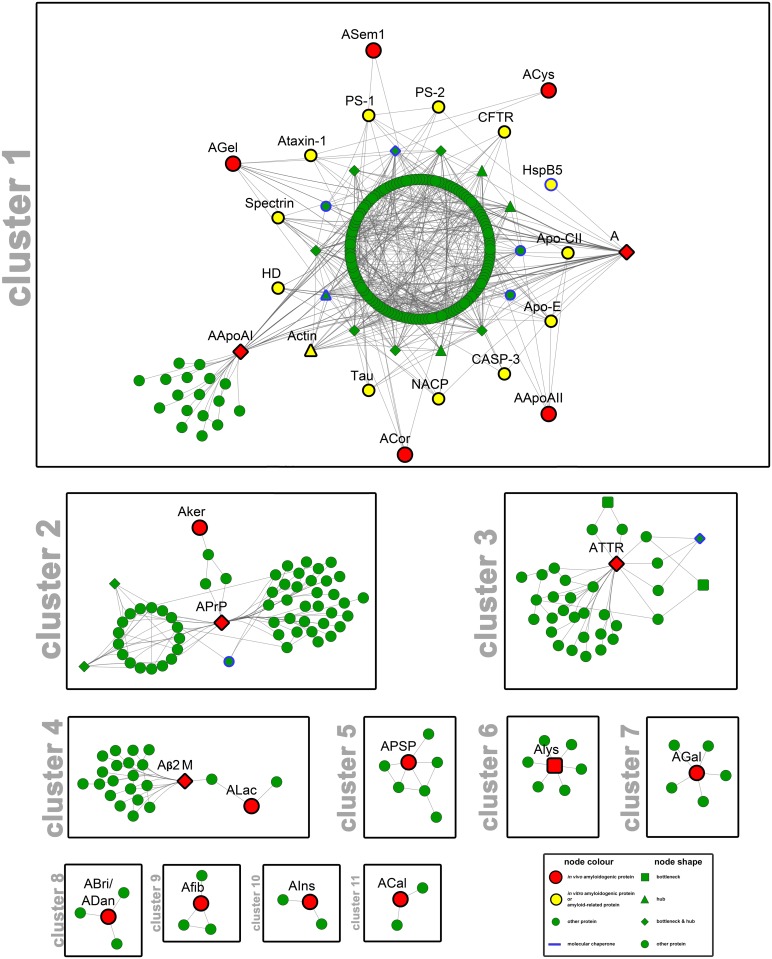
Clustering analysis of the amyloid interactome. The 11 clusters with 3 or more nodes of the amyloid interaction network, derived utilizing the MCL algorithm [97]. Cytoscape [51] was used as a visualization tool. The visual legend summarizes the shortcuts of node colour and node shape (See also Fig 1). The highly connected subnetwork of the first cluster within the amyloid interactome reveals the strong affinity between 7 amyloidogenic proteins (cluster 1—red nodes) and the integral representation of the proteins presented in Table 2 (cluster 1—yellow nodes) (Interactive cluster subnetworks available at http://83.212.109.111/amyloid_interactome).

The core of our study is the Amyloid Interactome, an interaction network represented as a large interconnected network with embedded functional sub-networks. Consequently, in order to further evaluate functional modules, a network clustering analysis was performed, utilizing the MCL algorithm [97]. The network was divided in 20 clusters, 11 of which composed of three or more nodes (Fig 2), while 9 contained only two proteins and were not further analyzed. The inflation value of 1.8 allowed the creation of compact clusters, preventing the network’s fragmentation.

The most important cluster, retrieved after the MCL implementation, consists of 186 protein-nodes (Fig 2, cluster 1). The results revealed the strong association between 7 *in vivo* amyloidogenic proteins (Fig 2, cluster 1—red nodes) and 13 proteins related to amyloid fibril formation (Fig 2, cluster 1—yellow nodes). Impressively, the full list of proteins included in the amyloid interactome and recorded as *in vitro* amyloid forming proteins or proteins related to amyloid fibril formation (Table 2), is solidly represented in this first cluster. This finding further validates the strong interconnection observed in our initial interaction network (Fig 1), implying that all these 13 proteins are likely important determinants of protein aggregation.

Notably, the most important subgroup of our interaction network (Fig 2, cluster 1) includes direct or indirect well-known amyloidogenic interaction partners of Aβ. Namely, amyloid forming Apolipoproteins (AApoAI, AApoAI*I*) are catalytic binding partners [98], whereas ACys [16, 99] and AGel [100, 101] prevent Aβ from accumulation. Moreover, Aβ cooperated with 9 (HspB5, Spectrin, Actin, PS-1, PS-2, ApoE, CASP-3, Tau, NACP) out of the 13 proteins of Table 2, both in the entire network and the fragmented network, a finding that might stimulate new ideas about the nature of Aβ interactions.

The amyloidogenic proteins, APrP and AKer are located in the second cluster, together with Clusterin (Fig 2, cluster 2—node with blue border), an extracellular chaperone, present in disease-associated extracellular amyloid deposits [102] and a proteasome functional subunit (Proteasome subunit alpha type-3). The absence of Clusterin from cluster 1, though, has prompted increasing interest, since the well-studied molecular chaperone is an existing protein target for the Alzheimer’s disease and so, we would expect a strong correlation with Aβ [103]. Nevertheless, MCL algorithm results point toward a possible relationship between Clusterin and Aker or APrp, a finding that remains to be elucidated. Additional proteins of this cluster are associated with transcriptional regulation and pre-mRNA splicing, since the over-expression of prions influences normal cellular proteins, participating in apoptosis or cell signaling [104]. Transthyretin, a potent inhibitor of Aβ [72], created a separate cluster together with Small ubiquitin-related modifier 3. The remaining complexes, consist of less than 7 nodes, where, with the exception of cluster 4, each one contains only one amyloidogenic protein (Fig 2, clusters 4–11).

#### Functional enrichment analysis

Functional interpretation of the data, derived from each cluster, was performed using BiNGO [57] and thus, statistically significant GO terms [59] were obtained for three functional categories (biological process, molecular function and cellular component). Due to the excess of information derived from this analysis, terms with great statistical and biological significance were manually selected to functionally characterize each cluster. Importantly, as mentioned before, similar subcategories with the entire network analysis resulted from the cluster functional analysis, and “response to stress” was the most significant function in the majority of the clusters (details of cluster 1 GO enrichment are shown in S6 Table). Therefore, it is apparent from all the above results that the amyloid interaction map locates in “spatial proximity” proteins related to stress (chaperones, co-chaperones and amyloidogenic proteins), which arise as a response to pathological conditions [88] (S4B Fig). Nevertheless, biological systems are dynamic, meaning that a complex succession of events may occur over the course of time, in contrast with a protein-protein interaction network. Therefore, certain events described on the amyloid interactome are based on a static system and thus, this analysis could produce certain artificial results that should be addressed carefully to draw biologically significant conclusions.

#### Pathway analysis and disease association

KEGG pathway analysis was performed, in an attempt to detect common metabolic pathways, in which the network’s proteins participate. A complex series of signaling pathways including the MAPK signaling pathway, B-cell and T-cell signaling pathways and the insulin signaling pathway are associated with the network’s proteins. Additional disease association analysis, conducted with WebGestalt [105], revealed significant associated disorders for every cluster. In the first cluster (Fig 2), for example, Tauopathies, Dementia and Alzheimer’s disease constitute the most significant group of pathologies. Pinpointing the components of such disease pathways is a promising perspective and thus, a detailed analysis and a novel joined network of diseases related to amyloidoses is currently being under construction (research article in preparation).

It is important to note that a delicate feature of our interactome, and its subsequent fragmentation, is the vast amount of experimental data on Aβ peptide polymerization, owing to the worldwide prevalence of the Alzheimer’s disease and the shortage of data on other less studied amyloidogenic proteins.

### Rational design of protein inhibitors

**Fig 3:**
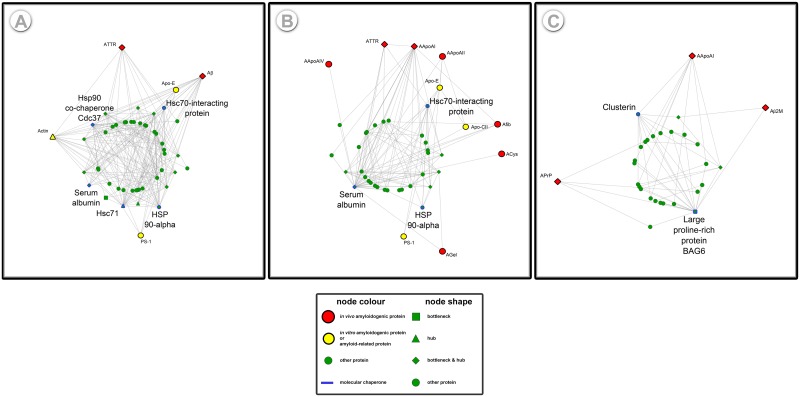
Subnetworks of molecular chaperones participating in the amyloid interactome. 3 important subnetworks were isolated from the entire amyloid interactome: (A) Subnetwork of Hsp90 co-chaperone Cdc37, Hsc70-interacting protein, Hsp 90-alpha, Hsc71 and their first neighbors, (B) Subnetwork of Serum albumin and Hsc70-interacting protein and their first neighbors and (C) Subnetwork of Clusterin, Large proline-rich protein BAG6 and their first neighbors. The aforementioned proteins, having chaperone or co-chaperone activity, were found to play a pivotal role in the integrity of the interactome (See section Network Analysis Based on Graph Theory). A highly selective and direct correlation of Serum albumin and 6 amyloidogenic proteins was observed (B), whereas indirect interactions between Serum albumin and 2 amyloidogenic proteins were recorded (A). Hsc70-interacting protein is a significant element of the interactome, since it conciliates interactions between Apolipoproteins and ACys or ATTR (A,B). Clusterin synergistically with Large proline-rich protein BAG6 interferes with APrp and Aβ2M (C). The finding that more than one chaperones mediate the interconnection between different amyloidogenic proteins deserves further investigation.

Moving a step forward it seems interesting to investigate the correlation between biological factors, participating in the amyloid interactome that could influence amyloidogenicity. To determine whether significant elements of the amyloid interactome might indicate common properties of good candidates to be targeted by therapy, hubs and bottlenecks were thoroughly examined (Fig 3).

An important group of proteins, dynamically participating in the interactome, is the group of molecular chaperones (S3D Fig). Their active role in controlling protein aggregation and their close relation to amyloidogenic proteins is the “hidden weapon" of the protein machinery and the reason why 4 out of 9 chaperones were recorded as hubs and bottlenecks in the amyloid interactome (Fig 1). The transient nature of real-time interactions between chaperones and their partners, though, results in unrealistic low connectivity, in signaling and mitochondrial protein–protein interaction networks [106].

Contradictory theories on whether chaperones are “guilty or innocent” during the protein aggregation process provoke an intense debate. In particular, several experimental studies revealed the co-localization of chaperones with various amyloidogenic proteins [107, 108], whereas other experimental work reported on the inhibitory properties of chaperones, when added during fibrillation [102, 109, 110]. Impressively, molecular chaperones have been shown to inhibit the formation of amyloid fibrils even when present at extremely sub-stoichiometric ratios, comparing to amyloid forming protein [111]. Fig 3 illustrates the interaction partners of selected molecular chaperones, prospect for the rational design of aggregation inhibitors. All characteristic path lengths for the subnetworks shown in Fig 3 are reduced approximately by one degree, in comparison with those of the entire amyloid interactome (CPL ~ 2.4). This result quantifies the great importance of chaperones as mediators of communication between amyloidogenic proteins.

Plasma proteins, on the other hand, is another promising group of protein-targets, considering that it includes proteins with many interaction partners, which have a wide range of physiological functions. Noteworthy, this group of proteins contains significant amyloidogenic components of the amyloid interactome, such as ATTR and Aβ2M. Among them, Serum albumin is a co-chaperone with significant topological features in the amyloid interactome, since it acts both as a hub and a bottleneck (Fig 3A and 3B). As Fig 3 illustrates, Serum albumin interacts directly or indirectly with *in vivo* amyloidogenic proteins and proteins related to amyloid fibril formation (See Fig 3 legend for details).

Normally, Albumin is a prevalent transporter of human plasma, known to carry a wide range of molecules, but under *in vitro* conditions was found to self-assemble into typical amyloid fibrils [112]. Nevertheless, it is worth mentioning that Serum Albumin was absent from our initial non-redundant list of amyloidogenic proteins (S1 Table), since such an entry was not found recorded neither in our literature sources nor deposited in AmyLoad or UniProtKB. Despite the “aggregation-prone” nature of Albumin, the presence of this plasma protein promotes neuronal survival [113] or inhibits amyloid fibrillation in *in vitro* designed experiments [114], while according to the proteomic analysis, conducted by Hye et al., is an eligible biomarker for the Alzheimer’s Disease [115]. Therefore, because of its central role in the interactome (Fig 3A and 3B) and the previously recorded features as a potent inhibitor of fibrillation, it seems that human plasma Albumin is a challenging molecule, which might stimulate new ideas about the design of anti-amyloid drugs.

From the above discussion, we pinpointed hubs as the most competent candidates regarding the therapeutic intervention of amyloidogenicity. Opposing studies, though, suggest that proteins with low connectivity would be more efficient therapeutic targets for neurodegenerative diseases, since hubs are generally considered as “elegant features” for the robustness of an interactome [35]. Therefore, our computational approach should be followed by a variety of *in vitro*, cellular and *in vivo* experiments, in order to verify our speculations.

## Conclusions

Given the complexity of the molecular mechanisms driving amyloid fibrillation, a frequently used strategy is directed towards studying amyloidogenicity at molecular level, although, this approach is not always feasible, due to the dynamics of protein misfolding. In this study, we attempted to answer the crucial question of amyloidogenicity, following the principles of systems biology, by assembling a group of “miscellaneous” proteins into a common biological interaction network. The amyloid interactome illustrates a united interaction network of critical hypotheses, regarding the irregular protein aggregation, since it represents an integrated protein map of *in vivo* amyloidogenic proteins, together with *in vitro* amyloid forming proteins or proteins related to amyloid fibril formation. The amyloidogenic and non-amyloidogenic elements of the amyloid interactome eventually come together to form a complex “tapestry” of protein-protein interactions. Based on the complex network theory analysis, this network exhibits topological properties that are similar to other significant interaction networks. Surprisingly, our approach, apart from validating all previously experimentally verified direct or indirect protein interactions, allowed us to stress the importance of novel protein targets. Our integrated project has raised plentiful questions and could serve as the driving force to guide the experimental process in the challenging field of protein aggregation, even at the molecular level. Nevertheless, it should be addressed that the amyloid interactome was built based on the current knowledge of protein-protein interactions, meaning that there is a “publication bias” between over and understudied amyloidogenic proteins. Individual proteins should be carefully validated, utilizing the appropriate methodology, in order to enhance the significance of our observations. Therefore, the strength of the amyloid interactome lies in the perspective to identify key mediators of amyloidogenicity that could be targeted therapeutically.

## Supporting information

S1 Fig Study design workflow of the amyloid interactome. An overview of the basic protocol, used to create and analyze the amyloid interactome.
(TIF)

S2 Fig Distributions for three complex topological parameters of the amyloid interactome and results from “Lethality Testing”. **(A) Node degree distribution** in log-log plot. The red line shows that the distribution decays as a power law (*P(k) = 117*.*86k*^*-1*.*236*^). Nodes on the upper left corner of the chart (high node degree) are hubs in the amyloid interactome. **(B) Betweenness centrality distribution** with the horizontal axis in a logarithmic scale. Nodes on the right quarter of the chart (high betweeness centrality) are bottlenecks in the network. **(C) Average clustering coefficient distribution.** The red line shows that it follows approximately the scaling law (*C(k) = 0*.*816k*^*-0*.*647*^), designating the network’s ability to form clusters. **(D) Lethality testing.** This chart shows the effect of the gradual removal of random nodes (blue circles) and the gradual removal of hubs (black triangles) and bottlenecks (grey squares), on the Characteristic Path Length (CPL) of the network (For detailed discussion please refer to Results and discussion section).
(TIF)

S3 Fig Detailed features of the amyloid interactome. **(A)** Interactions between *in vivo* amyloidogenic proteins (red-coloured nodes) and *in vitro* amyloid forming proteins or proteins related to amyloid fibril formation (yellow-coloured nodes). **(B)** Yellow-coloured nodes represent *in vitro* amyloid forming proteins or proteins related to amyloid fibril formation and are a delicate feature of the amyloid interactome. **(C)** Representation of the key role of non-amyloidogenic hubs and bottlenecks in the amyloid interactome. Triangles are proteins acting as hubs, squares are proteins acting as bottlenecks and diamonds are proteins acting as both. **(D)** Nodes with blue borders represent proteins characterized as chaperones or co-chaperones (Interactive network available at http://83.212.109.111/amyloid_interactome).
(TIF)

S4 Fig GO functional analysis of enriched terms in the biological process ontology for the entire amyloid interactome and the first cluster. Functionally grouped networks of enriched categories were generated both for the amyloid interactome (A) and cluster 1 (B). GO terms are represented as nodes. The colour gradient of each circle corresponds to the p-value of the associated GO term. White-coloured nodes are not statistically significant nodes, but are parent nodes of statistically significant GO terms. Different node sizes are indicative of varying frequencies of the proteins correlated with each GO term (See S4 and S6 Tables).
(TIF)

S1 Table The non-redundant, detailed catalogue of *in vivo* and *in vitro* amyloidogenic proteins or peptide fragments or proteins related to amyloid fibril formation. This extended list of proteins includes proteins known to self-assemble into typical amyloid fibrils *in vivo*, along with intracellular inclusions with known biochemical composition, published by Sipe *et al*. in 2014 [2]. In addition to this, the list includes proteins which form amyloid fibrils *in vitro* [45], protein segments obtained from AmyLoad [5] and finally, UniProtKB entries [46] of proteins related to amyloid fibril formation. A UniProtKB Accession Number is provided for each protein. The original source library of each protein is tagged with a cross (+). The Digital Object Identifier (DOI) code is provided, when it is available (See Materials and methods).
(PDF)

S2 Table The top 20 hubs of the amyloid interactome. The 20 proteins with the highest node degrees are considered as hubs in the amyloid interactome. 6 of these proteins belong to the dataset of the amyloidogenic proteins, described in S1 Table, whilst the rest of the hubs exhibit numerous functions, acting mainly as chaperones, signal transducers or structural constituent of the cell (See Results and discussion).
(PDF)

S3 Table The top 20 bottlenecks of the amyloid interactome. The 20 proteins with the highest betweeness centralities are considered bottlenecks in the amyloid interactome. 6 of these proteins belong to the dataset of the amyloidogenic proteins, described in S1 Table. 15 bottlenecks exhibit high node degree values and are also considered as hubs in this network (S2 Table).
(PDF)

S4 Table Amyloid interactome GO term enrichment. A p-value of 10E-14 was set as a gathering threshold for Biological Process and Cellular Component, whereas a value of 10E-8 was set as threshold for Molecular Function. Proteins of the entire amyloid interactome were subjected to a GO term enrichment analysis using BiNGO [56]. The UniProtKB ACs of proteins that are characterized by overrepresented GO terms in the entire amyloid interactome are given in this table, along with their number and their frequency in the network. The adjusted p-value suggests the importance of these GO terms in the proteins of the amyloid interactome.
(PDF)

S5 Table GO Terms enrichment of the 18 important, non-amyloidogenic hubs and bottlenecks identified on the amyloid interactome. The majority of proteins, which are characterised as hubs and bottlenecks are involved in signal transduction and in several metabolic processes. The most abundant GO term, though, is response to stress, in accordance with the most represented group of the amyloid interactome (See S4 Table).
(PDF)

S6 Table Enriched GO categories of Cluster 1, derived from the amyloid interactome. Enriched categories for Biological Process are those with p<10E-12, for Cellular Component with p<10E-14 and for Molecular Function with p<10E-8. Proteins of the first cluster of the amyloid interactome were subjected to a GO term enrichment analysis using BiNGO [56]. The UniProtKB ACs of proteins that are characterized by overrepresented GO terms in this cluster are given in this table, along with their number and their frequency in the network. The adjusted p-value suggests the importance of these GO terms in the proteins of cluster 1.
(PDF)

S1 File Web application user guide. Detailed description of the structure of the interactive Amyloid Interactome–Web application is available at http://83.212.109.111/amyloid_interactome.
(PDF)

## References

[1] Fandrich M. On the structural definition of amyloid fibrils and other polypeptide aggregates. Cellular and molecular life sciences: CMLS. 2007;64(16):2066–78. 10.1007/s00018-007-7110-2 17530168 PMC11138455

[2] Sipe JD, Benson MD, Buxbaum JN, Ikeda S, Merlini G, Saraiva MJ, et al Nomenclature 2014: Amyloid fibril proteins and clinical classification of the amyloidosis. Amyloid: the international journal of experimental and clinical investigation: the official journal of the International Society of Amyloidosis. 2014;21(4):221–4. 10.3109/13506129.2014.964858 25263598

[3] Chiti F, Webster P, Taddei N, Clark A, Stefani M, Ramponi G, et al Designing conditions for in vitro formation of amyloid protofilaments and fibrils. Proceedings of the National Academy of Sciences of the United States of America. 1999;96(7):3590–4. 10097081 10.1073/pnas.96.7.3590 PMC22338

[4] Harrison RS, Sharpe PC, Singh Y, Fairlie DP. Amyloid peptides and proteins in review. Reviews of physiology, biochemistry and pharmacology. 2007;159:1–77. 10.1007/112_2007_0701 17846922

[5] Bergman P, Roan NR, Romling U, Bevins CL, Munch J. Amyloid formation: functional friend or fearful foe? Journal of internal medicine. 2016;280(2):139–52. 10.1111/joim.12479 27151743 PMC4956559

[6] Iconomidou VA, Vriend G, Hamodrakas SJ. Amyloids protect the silkmoth oocyte and embryo. FEBS letters. 2000;479(3):141–5. 10981723 10.1016/s0014-5793(00)01888-3

[7] Wozniak PP, Kotulska M. AmyLoad: website dedicated to amyloidogenic protein fragments. Bioinformatics. 2015;31(20):3395–7. 10.1093/bioinformatics/btv375 26088800

[8] Pawlicki S, Le Bechec A, Delamarche C. AMYPdb: a database dedicated to amyloid precursor proteins. BMC bioinformatics. 2008;9:273 10.1186/1471-2105-9-273 18544157 PMC2442844

[9] Beerten J, Van Durme J, Gallardo R, Capriotti E, Serpell L, Rousseau F, et al WALTZ-DB: a benchmark database of amyloidogenic hexapeptides. Bioinformatics. 2015;31(10):1698–700. 10.1093/bioinformatics/btv027 25600945

[10] Thompson MJ, Sievers SA, Karanicolas J, Ivanova MI, Baker D, Eisenberg D. The 3D profile method for identifying fibril-forming segments of proteins. Proceedings of the National Academy of Sciences of the United States of America. 2006;103(11):4074–8. 10.1073/pnas.0511295103 16537487 PMC1449648

[11] Goldschmidt L, Teng PK, Riek R, Eisenberg D. Identifying the amylome, proteins capable of forming amyloid-like fibrils. Proceedings of the National Academy of Sciences of the United States of America. 2010;107(8):3487–92. 10.1073/pnas.0915166107 20133726 PMC2840437

[12] Uversky V, Talapatra A, Gillespie JR, Fink AL. Protein deposits as the molecular basis of amyloidosis. Part I. Systemic amyloidoses. Medical Science Monitor. 1999;5(5):1001–12.

[13] Uversky V, Talapatra A, Gillespie JR, Fink AL. Protein deposits as the molecular basis of amyloidosis. Part II. Localized amyloidosis and neurodegenerative disorders. Medical Science Monitor. 1999;5(6):1238–54.

[14] Bergstrom J, Murphy CL, Weiss DT, Solomon A, Sletten K, Hellman U, et al Two different types of amyloid deposits—apolipoprotein A-IV and transthyretin—in a patient with systemic amyloidosis. Laboratory investigation; a journal of technical methods and pathology. 2004;84(8):981–8. 10.1038/labinvest.3700124 15146166

[15] de Sousa MM, Vital C, Ostler D, Fernandes R, Pouget-Abadie J, Carles D, et al Apolipoprotein AI and transthyretin as components of amyloid fibrils in a kindred with apoAI Leu178His amyloidosis. The American journal of pathology. 2000;156(6):1911–7. 10.1016/S0002-9440(10)65064-X 10854214 PMC1850097

[16] Levy E, Sastre M, Kumar A, Gallo G, Piccardo P, Ghetti B, et al Codeposition of cystatin C with amyloid-beta protein in the brain of Alzheimer disease patients. Journal of neuropathology and experimental neurology. 2001;60(1):94–104. 11202179 10.1093/jnen/60.1.94

[17] Morales R, Moreno-Gonzalez I, Soto C. Cross-seeding of misfolded proteins: implications for etiology and pathogenesis of protein misfolding diseases. PLoS pathogens. 2013;9(9):e1003537 10.1371/journal.ppat.1003537 24068917 PMC3777858

[18] Tsigelny IF, Crews L, Desplats P, Shaked GM, Sharikov Y, Mizuno H, et al Mechanisms of hybrid oligomer formation in the pathogenesis of combined Alzheimer's and Parkinson's diseases. PloS one. 2008;3(9):e3135 10.1371/journal.pone.0003135 18769546 PMC2519786

[19] Guo JP, Arai T, Miklossy J, McGeer PL. Abeta and tau form soluble complexes that may promote self aggregation of both into the insoluble forms observed in Alzheimer's disease. Proceedings of the National Academy of Sciences of the United States of America. 2006;103(6):1953–8. 10.1073/pnas.0509386103 16446437 PMC1413647

[20] Morales R, Estrada LD, Diaz-Espinoza R, Morales-Scheihing D, Jara MC, Castilla J, et al Molecular cross talk between misfolded proteins in animal models of Alzheimer's and prion diseases. The Journal of neuroscience: the official journal of the Society for Neuroscience. 2010;30(13):4528–35. 20357103 10.1523/JNEUROSCI.5924-09.2010 PMC2859074

[21] Yan J, Fu X, Ge F, Zhang B, Yao J, Zhang H, et al Cross-seeding and cross-competition in mouse apolipoprotein A-II amyloid fibrils and protein A amyloid fibrils. The American journal of pathology. 2007;171(1):172–80. 10.2353/ajpath.2007.060576 17591964 PMC1941612

[22] Westermark GT, Westermark P. Transthyretin and amyloid in the islets of Langerhans in type-2 diabetes. Experimental diabetes research. 2008;2008:429274 10.1155/2008/429274 18825272 PMC2553203

[23] Ulbrich L, Cozzolino M, Marini ES, Amori I, De Jaco A, Carri MT, et al Cystatin B and SOD1: protein-protein interaction and possible relation to neurodegeneration. Cellular and molecular neurobiology. 2014;34(2):205–13. 10.1007/s10571-013-0004-y 24234043 PMC11488905

[24] Gotz J, Chen F, van Dorpe J, Nitsch RM. Formation of neurofibrillary tangles in P301l tau transgenic mice induced by Abeta 42 fibrils. Science. 2001;293(5534):1491–5. 10.1126/science.1062097 11520988

[25] Levy E. Cystatin C: a potential target for Alzheimer's treatment. Expert review of neurotherapeutics. 2008;8(5):687–9. 10.1586/14737175.8.5.687 18457524

[26] Cusick ME, Klitgord N, Vidal M, Hill DE. Interactome: gateway into systems biology. Human molecular genetics. 2005;14 Spec No. 2:R171–81. 16162640 10.1093/hmg/ddi335

[27] Bader S, Kuhner S, Gavin AC. Interaction networks for systems biology. FEBS letters. 2008;582(8):1220–4. 10.1016/j.febslet.2008.02.015 18282471

[28] Stelzl U, Worm U, Lalowski M, Haenig C, Brembeck FH, Goehler H, et al A human protein-protein interaction network: a resource for annotating the proteome. Cell. 2005;122(6):957–68. 10.1016/j.cell.2005.08.029 16169070

[29] Rhodes DR, Chinnaiyan AM. Integrative analysis of the cancer transcriptome. Nature genetics. 2005;37 Suppl:S31–7. 15920528 10.1038/ng1570

[30] Gunsalus KC, Ge H, Schetter AJ, Goldberg DS, Han JD, Hao T, et al Predictive models of molecular machines involved in Caenorhabditis elegans early embryogenesis. Nature. 2005;436(7052):861–5. 10.1038/nature03876 16094371

[31] Rual JF, Venkatesan K, Hao T, Hirozane-Kishikawa T, Dricot A, Li N, et al Towards a proteome-scale map of the human protein-protein interaction network. Nature. 2005;437(7062):1173–8. 10.1038/nature04209 16189514

[32] Ge H, Walhout AJ, Vidal M. Integrating 'omic' information: a bridge between genomics and systems biology. Trends in genetics: TIG. 2003;19(10):551–60. 10.1016/j.tig.2003.08.009 14550629

[33] Petta I, Lievens S, Libert C, Tavernier J, De Bosscher K. Modulation of Protein-Protein Interactions for the Development of Novel Therapeutics. Molecular therapy: the journal of the American Society of Gene Therapy. 2016;24(4):707–18. 26675501 10.1038/mt.2015.214 PMC4886928

[34] Chen JY, Shen C, Sivachenko AY. Mining Alzheimer disease relevant proteins from integrated protein interactome data. Pacific Symposium on Biocomputing Pacific Symposium on Biocomputing. 2006:367–78. 17094253

[35] Goni J, Esteban FJ, de Mendizabal NV, Sepulcre J, Ardanza-Trevijano S, Agirrezabal I, et al A computational analysis of protein-protein interaction networks in neurodegenerative diseases. BMC systems biology. 2008;2:52 10.1186/1752-0509-2-52 18570646 PMC2443111

[36] Rao VS, Srinivas K, Sujini GN, Kumar GN. Protein-protein interaction detection: methods and analysis. International journal of proteomics. 2014;2014:147648 10.1155/2014/147648 24693427 PMC3947875

[37] Tourette C, Li B, Bell R, O'Hare S, Kaltenbach LS, Mooney SD, et al A large scale Huntingtin protein interaction network implicates Rho GTPase signaling pathways in Huntington disease. The Journal of biological chemistry. 2014;289(10):6709–26. 10.1074/jbc.M113.523696 24407293 PMC3945331

[38] Bai Y, Markham K, Chen F, Weerasekera R, Watts J, Horne P, et al The in vivo brain interactome of the amyloid precursor protein. Molecular & cellular proteomics: MCP. 2008;7(1):15–34. 17934213 10.1074/mcp.M700077-MCP200

[39] Silva JV, Yoon S, Domingues S, Guimaraes S, Goltsev AV, da Cruz ESEF, et al Amyloid precursor protein interaction network in human testis: sentinel proteins for male reproduction. BMC bioinformatics. 2015;16:12 10.1186/s12859-014-0432-9 25591988 PMC4384327

[40] Virok DP, Simon D, Bozso Z, Rajko R, Datki Z, Balint E, et al Protein array based interactome analysis of amyloid-beta indicates an inhibition of protein translation. Journal of proteome research. 2011;10(4):1538–47. 10.1021/pr1009096 21244100

[41] Harrison RS, Sharpe PC, Singh Y, Fairlie DP. Amyloid peptides and proteins in review In: Amara SG, Bamberg E, Fleischmann B, Gudermann T, Hebert SC, Jahn R, et al, editors. Reviews of physiology, biochemistry and pharmacology. Berlin, Heidelberg: Springer Berlin Heidelberg; 2007 p. 1–77. 10.1007/112_2007_0701 17846922

[42] UniProt C. UniProt: a hub for protein information. Nucleic acids research. 2015;43(Database issue):D204–12. 10.1093/nar/gku989 25348405 PMC4384041

[43] D'Souza A, Theis JD, Vrana JA, Dogan A. Pharmaceutical amyloidosis associated with subcutaneous insulin and enfuvirtide administration. Amyloid: the international journal of experimental and clinical investigation: the official journal of the International Society of Amyloidosis. 2014;21(2):71–5. 10.3109/13506129.2013.876984 PMC4021035 24446896

[44] Isobe T. AA amyloidosis and AL amyloidosis. Internal medicine. 1993;32(12):919–20. 8204969 10.2169/internalmedicine.32.919

[45] Hermjakob H, Montecchi-Palazzi L, Lewington C, Mudali S, Kerrien S, Orchard S, et al IntAct: an open source molecular interaction database. Nucleic acids research. 2004;32(Database issue):D452–5. 10.1093/nar/gkh052 14681455 PMC308786

[46] Stark C, Breitkreutz BJ, Reguly T, Boucher L, Breitkreutz A, Tyers M. BioGRID: a general repository for interaction datasets. Nucleic acids research. 2006;34(Database issue):D535–9. 10.1093/nar/gkj109 16381927 PMC1347471

[47] von Mering C, Huynen M, Jaeggi D, Schmidt S, Bork P, Snel B. STRING: a database of predicted functional associations between proteins. Nucleic acids research. 2003;31(1):258–61. Epub 2003/01/10. 12519996 10.1093/nar/gkg034 PMC165481

[48] Licata L, Orchard S. The MIntAct Project and Molecular Interaction Databases. Methods in molecular biology. 2016;1415:55–69. 10.1007/978-1-4939-3572-7_3 27115627

[49] Kerrien S, Orchard S, Montecchi-Palazzi L, Aranda B, Quinn AF, Vinod N, et al Broadening the horizon—level 2.5 of the HUPO-PSI format for molecular interactions. BMC biology. 2007;5:44 10.1186/1741-7007-5-44 17925023 PMC2189715

[50] Nastou KC, Tsaousis GN, Kremizas KE, Litou ZI, Hamodrakas SJ. The human plasma membrane peripherome: visualization and analysis of interactions. BioMed research international. 2014;2014:397145 10.1155/2014/397145 25057483 PMC4095733

[51] Shannon P, Markiel A, Ozier O, Baliga NS, Wang JT, Ramage D, et al Cytoscape: a software environment for integrated models of biomolecular interaction networks. Genome research. 2003;13(11):2498–504. 10.1101/gr.1239303 14597658 PMC403769

[52] Lotia S, Montojo J, Dong Y, Bader GD, Pico AR. Cytoscape app store. Bioinformatics. 2013;29(10):1350–1. Epub 2013/04/19. 10.1093/bioinformatics/btt138 23595664 PMC3654709

[53] Assenov Y, Ramirez F, Schelhorn SE, Lengauer T, Albrecht M. Computing topological parameters of biological networks. Bioinformatics. 2008;24(2):282–4. 10.1093/bioinformatics/btm554 18006545

[54] Franz M, Lopes CT, Huck G, Dong Y, Sumer O, Bader GD. Cytoscape.js: a graph theory library for visualisation and analysis. Bioinformatics. 2016;32(2):309–11. 10.1093/bioinformatics/btv557 26415722 PMC4708103

[55] Morris JH, Apeltsin L, Newman AM, Baumbach J, Wittkop T, Su G, et al clusterMaker: a multi-algorithm clustering plugin for Cytoscape. BMC bioinformatics. 2011;12:436 10.1186/1471-2105-12-436 22070249 PMC3262844

[56] Brohee S, van Helden J. Evaluation of clustering algorithms for protein-protein interaction networks. BMC bioinformatics. 2006;7:488 10.1186/1471-2105-7-488 17087821 PMC1637120

[57] Maere S, Heymans K, Kuiper M. BiNGO: a Cytoscape plugin to assess overrepresentation of gene ontology categories in biological networks. Bioinformatics. 2005;21(16):3448–9. 10.1093/bioinformatics/bti551 15972284

[58] Wang J, Duncan D, Shi Z, Zhang B. WEB-based GEne SeT AnaLysis Toolkit (WebGestalt): update 2013. Nucleic acids research. 2013;41(Web Server issue):W77–83. 10.1093/nar/gkt439 23703215 PMC3692109

[59] Ashburner M, Ball CA, Blake JA, Botstein D, Butler H, Cherry JM, et al Gene ontology: tool for the unification of biology. The Gene Ontology Consortium. Nature genetics. 2000;25(1):25–9. 10.1038/75556 10802651 PMC3037419

[60] Collavin L, Lunardi A, Del Sal G. p53-family proteins and their regulators: hubs and spokes in tumor suppression. Cell death and differentiation. 2010;17(6):901–11. 10.1038/cdd.2010.35 20379196

[61] Vinayagam A, Zirin J, Roesel C, Hu Y, Yilmazel B, Samsonova AA, et al Integrating protein-protein interaction networks with phenotypes reveals signs of interactions. Nature methods. 2014;11(1):94–9. 10.1038/nmeth.2733 24240319 PMC3877743

[62] Fukuyama H, Verdier Y, Guan Y, Makino-Okamura C, Shilova V, Liu X, et al Landscape of protein-protein interactions in Drosophila immune deficiency signaling during bacterial challenge. Proceedings of the National Academy of Sciences of the United States of America. 2013;110(26):10717–22. 10.1073/pnas.1304380110 23749869 PMC3696746

[63] Schwikowski B, Uetz P, Fields S. A network of protein-protein interactions in yeast. Nature biotechnology. 2000;18(12):1257–61. 10.1038/82360 11101803

[64] Rao L, Zhang IY, Guo W, Feng L, Meggers E, Xu X. Nonfitting protein-ligand interaction scoring function based on first-principles theoretical chemistry methods: development and application on kinase inhibitors. Journal of computational chemistry. 2013;34(19):1636–46. 10.1002/jcc.23303 23681957

[65] Carter DB. The interaction of amyloid-beta with ApoE. Sub-cellular biochemistry. 2005;38:255–72. 15709483 10.1007/0-387-23226-5_13

[66] Westermark P, Eriksson L, Engstrom U, Enestrom S, Sletten K. Prolactin-derived amyloid in the aging pituitary gland. The American journal of pathology. 1997;150(1):67–73. 9006323 PMC1858515

[67] Maji SK, Perrin MH, Sawaya MR, Jessberger S, Vadodaria K, Rissman RA, et al Functional amyloids as natural storage of peptide hormones in pituitary secretory granules. Science. 2009;325(5938):328–32. 10.1126/science.1173155 19541956 PMC2865899

[68] Wiltzius JJ, Sievers SA, Sawaya MR, Eisenberg D. Atomic structures of IAPP (amylin) fusions suggest a mechanism for fibrillation and the role of insulin in the process. Protein science: a publication of the Protein Society. 2009;18(7):1521–30. 19475663 10.1002/pro.145 PMC2775219

[69] Lu J, Yu Y, Zhu I, Cheng Y, Sun PD. Structural mechanism of serum amyloid A-mediated inflammatory amyloidosis. Proceedings of the National Academy of Sciences of the United States of America. 2014;111(14):5189–94. 10.1073/pnas.1322357111 24706838 PMC3986191

[70] Eriksen N, Benditt EP. Isolation and characterization of the amyloid-related apoprotein (SAA) from human high density lipoprotein. Proceedings of the National Academy of Sciences of the United States of America. 1980;77(11):6860–4. 6161374 10.1073/pnas.77.11.6860 PMC350390

[71] Fronczak A, Fronczak P, Holyst JA. Average path length in random networks. Physical review E, Statistical, nonlinear, and soft matter physics. 2004;70(5 Pt 2):056110 10.1103/PhysRevE.70.056110 15600695

[72] Velayudhan L, Killick R, Hye A, Kinsey A, Guntert A, Lynham S, et al Plasma transthyretin as a candidate marker for Alzheimer's disease. Journal of Alzheimer's disease: JAD. 2012;28(2):369–75. 10.3233/JAD-2011-110611 22002789

[73] Leclerc RD. Survival of the sparsest: robust gene networks are parsimonious. Molecular systems biology. 2008;4:213 10.1038/msb.2008.52 18682703 PMC2538912

[74] Xiao Fan W, Guanrong C. Complex networks: small-world, scale-free and beyond. IEEE Circuits and Systems Magazine. 2003;3(1):6–20.

[75] Li X, Chen H, Huang Z, Su H, Martinez JD. Global mapping of gene/protein interactions in PubMed abstracts: a framework and an experiment with P53 interactions. Journal of biomedical informatics. 2007;40(5):453–64. 10.1016/j.jbi.2007.01.001 17317333 PMC2047827

[76] Barabasi AL, Albert R. Emergence of scaling in random networks. Science. 1999;286(5439):509–12. 10521342 10.1126/science.286.5439.509

[77] Barabasi AL, Oltvai ZN. Network biology: understanding the cell's functional organization. Nature reviews Genetics. 2004;5(2):101–13. 10.1038/nrg1272 14735121

[78] Albert R, Jeong H, Barabasi AL. Error and attack tolerance of complex networks. Nature. 2000;406(6794):378–82. 10.1038/35019019 10935628

[79] Yook SH, Jeong H, Barabasi AL. Modeling the Internet's large-scale topology. Proceedings of the National Academy of Sciences of the United States of America. 2002;99(21):13382–6. 10.1073/pnas.172501399 12368484 PMC129681

[80] Doyle JC, Alderson DL, Li L, Low S, Roughan M, Shalunov S, et al The "robust yet fragile" nature of the Internet. Proceedings of the National Academy of Sciences of the United States of America. 2005;102(41):14497–502. 10.1073/pnas.0501426102 16204384 PMC1240072

[81] Jalili M, Askari Sichani O, Yu X. Optimal pinning controllability of complex networks: dependence on network structure. Physical review E, Statistical, nonlinear, and soft matter physics. 2015;91(1):012803 10.1103/PhysRevE.91.012803 25679653

[82] Joy MP, Brock A, Ingber DE, Huang S. High-betweenness proteins in the yeast protein interaction network. Journal of biomedicine & biotechnology. 2005;2005(2):96–103. 16046814 10.1155/JBB.2005.96 PMC1184047

[83] Ravasz E, Somera AL, Mongru DA, Oltvai ZN, Barabasi AL. Hierarchical organization of modularity in metabolic networks. Science. 2002;297(5586):1551–5. 10.1126/science.1073374 12202830

[84] Gillis J, Ballouz S, Pavlidis P. Bias tradeoffs in the creation and analysis of protein-protein interaction networks. Journal of proteomics. 2014;100:44–54. 10.1016/j.jprot.2014.01.020 24480284 PMC3972268

[85] Goh KI, Cusick ME, Valle D, Childs B, Vidal M, Barabasi AL. The human disease network. Proceedings of the National Academy of Sciences of the United States of America. 2007;104(21):8685–90. 10.1073/pnas.0701361104 17502601 PMC1885563

[86] Lopez de la Paz M, Serrano L. Sequence determinants of amyloid fibril formation. Proceedings of the National Academy of Sciences of the United States of America. 2004;101(1):87–92. 10.1073/pnas.2634884100 14691246 PMC314143

[87] Teng PK, Eisenberg D. Short protein segments can drive a non-fibrillizing protein into the amyloid state. Protein engineering, design & selection: PEDS. 2009;22(8):531–6. 10.1093/protein/gzp037 PMC2719503 19602569

[88] Huang L, Liu X, Cheng B, Huang K. How our bodies fight amyloidosis: effects of physiological factors on pathogenic aggregation of amyloidogenic proteins. Archives of biochemistry and biophysics. 2015;568:46–55. 10.1016/j.abb.2015.01.007 25615529

[89] Calero M, Rostagno A, Ghiso J. Search for amyloid-binding proteins by affinity chromatography. Methods in molecular biology. 2012;849:213–23. 10.1007/978-1-61779-551-0_15 22528093 PMC3665336

[90] Gunawardana CG, Mehrabian M, Wang X, Mueller I, Lubambo IB, Jonkman JE, et al The Human Tau Interactome: Binding to the Ribonucleoproteome, and Impaired Binding of the Proline-to-Leucine Mutant at Position 301 (P301L) to Chaperones and the Proteasome. Molecular & cellular proteomics: MCP. 2015;14(11):3000–14. 26269332 10.1074/mcp.M115.050724 PMC4638042

[91] Carver JA, Rekas A, Thorn DC, Wilson MR. Small heat-shock proteins and clusterin: intra- and extracellular molecular chaperones with a common mechanism of action and function? IUBMB life. 2003;55(12):661–8. 10.1080/15216540310001640498 14769002

[92] Landreh M, Rising A, Presto J, Jornvall H, Johansson J. Specific chaperones and regulatory domains in control of amyloid formation. The Journal of biological chemistry. 2015;290(44):26430–6. 10.1074/jbc.R115.653097 26354437 PMC4646301

[93] Ellis RJ, van der Vies SM. Molecular chaperones. Annual review of biochemistry. 1991;60:321–47. 10.1146/annurev.bi.60.070191.001541 1679318

[94] Yerbury JJ, Kumita JR. Protein chemistry of amyloid fibrils and chaperones: implications for amyloid formation and disease. Current Chemical Biology. 2010;4(2):89–98.

[95] Soti C, Csermely P. Aging cellular networks: chaperones as major participants. Experimental gerontology. 2007;42(1–2):113–9. 10.1016/j.exger.2006.05.017 16814508

[96] Palotai R, Szalay MS, Csermely P. Chaperones as integrators of cellular networks: changes of cellular integrity in stress and diseases. IUBMB life. 2008;60(1):10–8. 10.1002/iub.8 18379988

[97] van Dongen S. Graph clustering by flow simulation [PhD]: Universiteit Utrecht; 2000.

[98] Koudinov AR, Berezov TT, Kumar A, Koudinova NV. Alzheimer's amyloid beta interaction with normal human plasma high density lipoprotein: association with apolipoprotein and lipids. Clinica chimica acta; international journal of clinical chemistry. 1998;270(2):75–84. 9544446 10.1016/s0009-8981(97)00207-6

[99] Tizon B, Ribe EM, Mi W, Troy CM, Levy E. Cystatin C protects neuronal cells from amyloid-beta-induced toxicity. Journal of Alzheimer's disease: JAD. 2010;19(3):885–94. 10.3233/JAD-2010-1291 20157244 PMC2889175

[100] Chauhan VP, Ray I, Chauhan A, Wisniewski HM. Binding of gelsolin, a secretory protein, to amyloid beta-protein. Biochemical and biophysical research communications. 1999;258(2):241–6. 10.1006/bbrc.1999.0623 10329371

[101] Ray I, Chauhan A, Wegiel J, Chauhan VP. Gelsolin inhibits the fibrillization of amyloid beta-protein, and also defibrillizes its preformed fibrils. Brain research. 2000;853(2):344–51. 10640633 10.1016/s0006-8993(99)02315-x

[102] Yerbury JJ, Poon S, Meehan S, Thompson B, Kumita JR, Dobson CM, et al The extracellular chaperone clusterin influences amyloid formation and toxicity by interacting with prefibrillar structures. FASEB journal: official publication of the Federation of American Societies for Experimental Biology. 2007;21(10):2312–22. 17412999 10.1096/fj.06-7986com

[103] Wilhelmus MM, de Waal RM, Verbeek MM. Heat shock proteins and amateur chaperones in amyloid-Beta accumulation and clearance in Alzheimer's disease. Molecular neurobiology. 2007;35(3):203–16. 10.1007/s12035-007-0029-7 17917109 PMC2039847

[104] Antony H, Wiegmans AP, Wei MQ, Chernoff YO, Khanna KK, Munn AL. Potential roles for prions and protein-only inheritance in cancer. Cancer metastasis reviews. 2012;31(1–2):1–19. 10.1007/s10555-011-9325-9 22138778 PMC3315606

[105] Zhang B, Kirov S, Snoddy J. WebGestalt: an integrated system for exploring gene sets in various biological contexts. Nucleic acids research. 2005;33(Web Server issue):W741–8. 10.1093/nar/gki475 15980575 PMC1160236

[106] Csermely P, Korcsmaros T, Kovacs IA, Szalay MS, Soti C. Systems biology of molecular chaperone networks. Novartis Foundation symposium. 2008;291:45–54; discussion -8, 137–40. 10.1002/9780470754030.ch4 18575265

[107] Sherman MY, Goldberg AL. Cellular defenses against unfolded proteins: a cell biologist thinks about neurodegenerative diseases. Neuron. 2001;29(1):15–32. 11182078 10.1016/s0896-6273(01)00177-5

[108] Wilhelmus MM, Boelens WC, Kox M, Maat-Schieman ML, Veerhuis R, de Waal RM, et al Small heat shock proteins associated with cerebral amyloid angiopathy of hereditary cerebral hemorrhage with amyloidosis (Dutch type) induce interleukin-6 secretion. Neurobiology of aging. 2009;30(2):229–40. 10.1016/j.neurobiolaging.2007.06.001 17629591

[109] Arimon M, Grimminger V, Sanz F, Lashuel HA. Hsp104 targets multiple intermediates on the amyloid pathway and suppresses the seeding capacity of Abeta fibrils and protofibrils. Journal of molecular biology. 2008;384(5):1157–73. 10.1016/j.jmb.2008.09.063 18851977

[110] Kudva YC, Hiddinga HJ, Butler PC, Mueske CS, Eberhardt NL. Small heat shock proteins inhibit in vitro A beta(1–42) amyloidogenesis. FEBS letters. 1997;416(1):117–21. 9369246 10.1016/s0014-5793(97)01180-0

[111] Lee S, Carson K, Rice-Ficht A, Good T. Small heat shock proteins differentially affect Abeta aggregation and toxicity. Biochemical and biophysical research communications. 2006;347(2):527–33. 10.1016/j.bbrc.2006.06.128 16828710

[112] Taboada P, Barbosa S, Castro E, Mosquera V. Amyloid fibril formation and other aggregate species formed by human serum albumin association. The journal of physical chemistry B. 2006;110(42):20733–6. 10.1021/jp064861r 17048876

[113] Vega L, Arroyo AA, Tabernero A, Medina JM. Albumin-blunted deleterious effect of amyloid-beta by preventing the internalization of the peptide into neurons. Journal of Alzheimer's disease: JAD. 2009;17(4):795–805. 10.3233/JAD-2009-1093 19542622

[114] Dominguez-Prieto M, Velasco A, Vega L, Tabernero A, Medina JM. Aberrant Co-localization of Synaptic Proteins Promoted by Alzheimer's Disease Amyloid-beta Peptides: Protective Effect of Human Serum Albumin. Journal of Alzheimer's disease: JAD. 2016;55(1):171–82. 10.3233/JAD-160346 PMC5115610 27662292

[115] Hye A, Lynham S, Thambisetty M, Causevic M, Campbell J, Byers HL, et al Proteome-based plasma biomarkers for Alzheimer's disease. Brain: a journal of neurology. 2006;129(Pt 11):3042–50. 17071923 10.1093/brain/awl279

