# Supplementary figures and images for: The amyloid interactome: Exploring protein aggregation
Source: PLoS One. 2017 Mar 1;12(3):e0173163. doi: 10.1371/journal.pone.0173163 (PMC5383009; doi:10.1371/journal.pone.0173163)

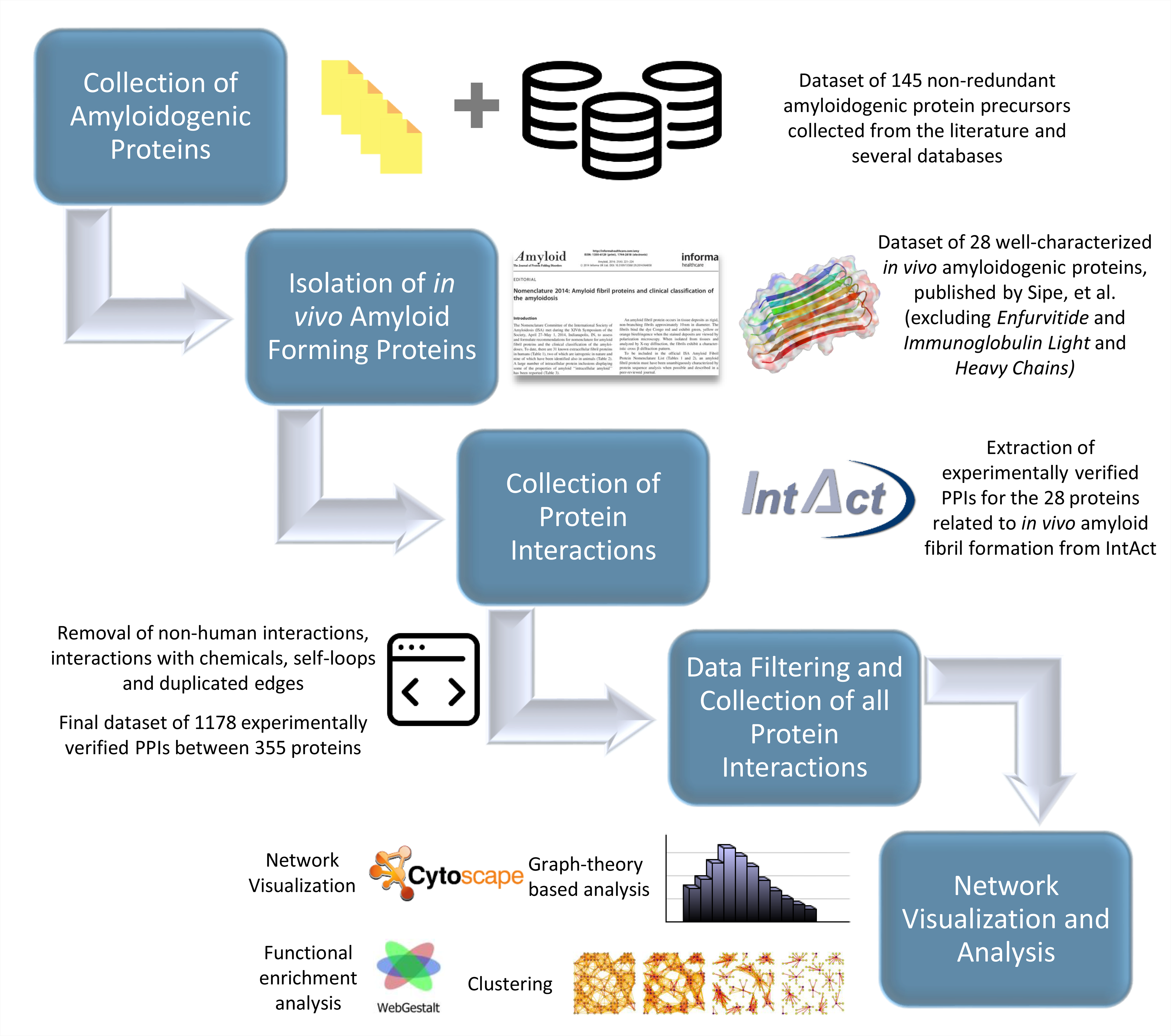

Supplement: S1 Fig — An overview of the basic protocol, used to create and analyze the amyloid interactome. (TIF) [file pone.0173163.s001.tif]

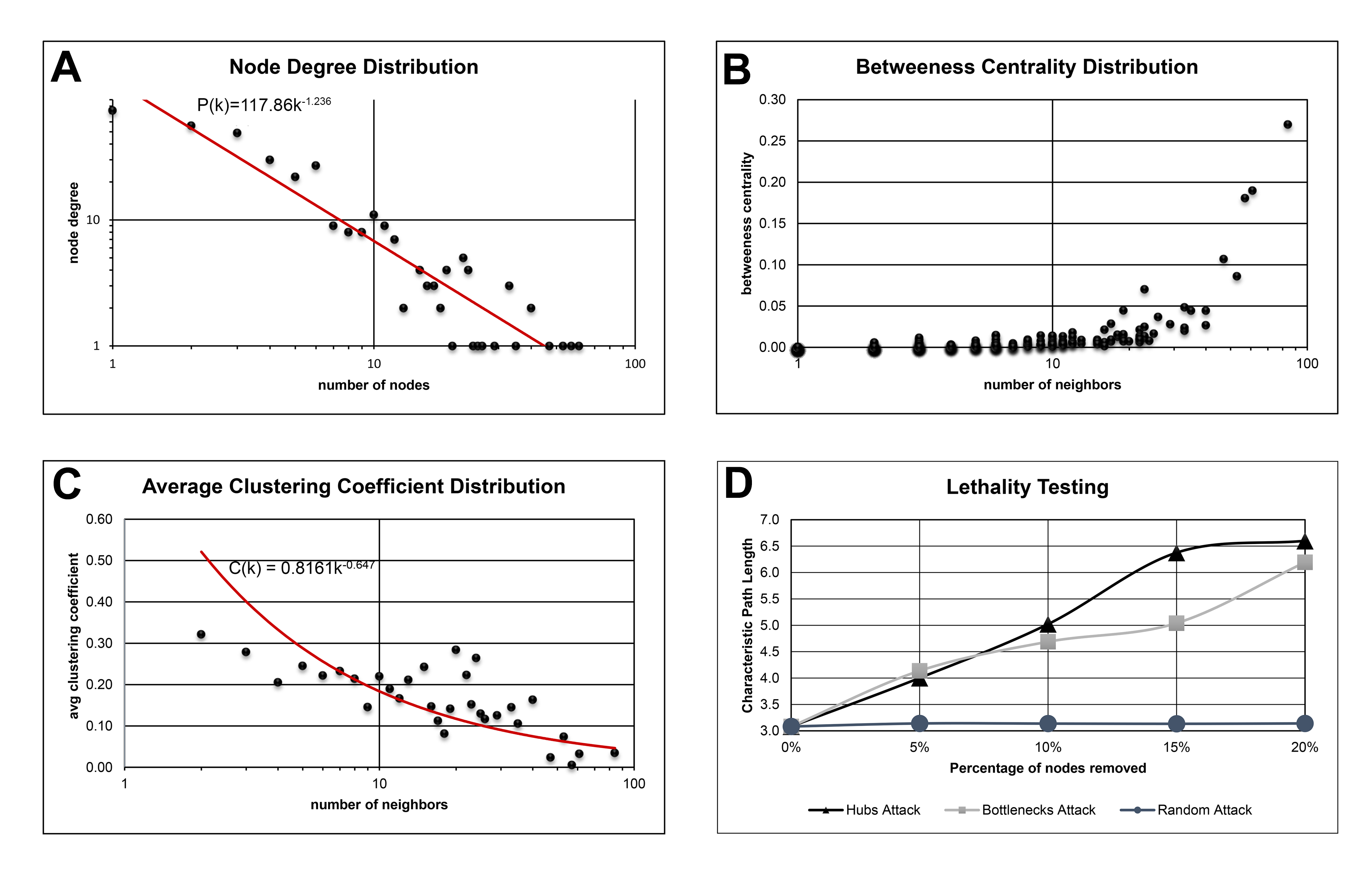

Supplement: S2 Fig — (A) Node degree distribution in log-log plot. The red line shows that the distribution decays as a power law (P(k) = 117.86k-1.236). Nodes on the upper left corner of the chart (high node degree) are hubs in the amyloid interactome. (B) Betweenness centrality distribution with the horizontal axis in a logarithmic scale. Nodes on the right quarter of the chart (high betweeness centrality) are bottlenecks in the network. (C) Average clustering coefficient distribution. The red line shows that it follows approximately the scaling law (C(k) = 0.816k-0.647), designating the network’s ability to form clusters. (D) Lethality testing. This chart shows the effect of the gradual removal of random nodes (blue circles) and the gradual removal of hubs (black triangles) and bottlenecks (grey squares), on the Characteristic Path Length (CPL) of the network (For detailed discussion please refer to Results and discussion section). (TIF) [file pone.0173163.s002.tif]

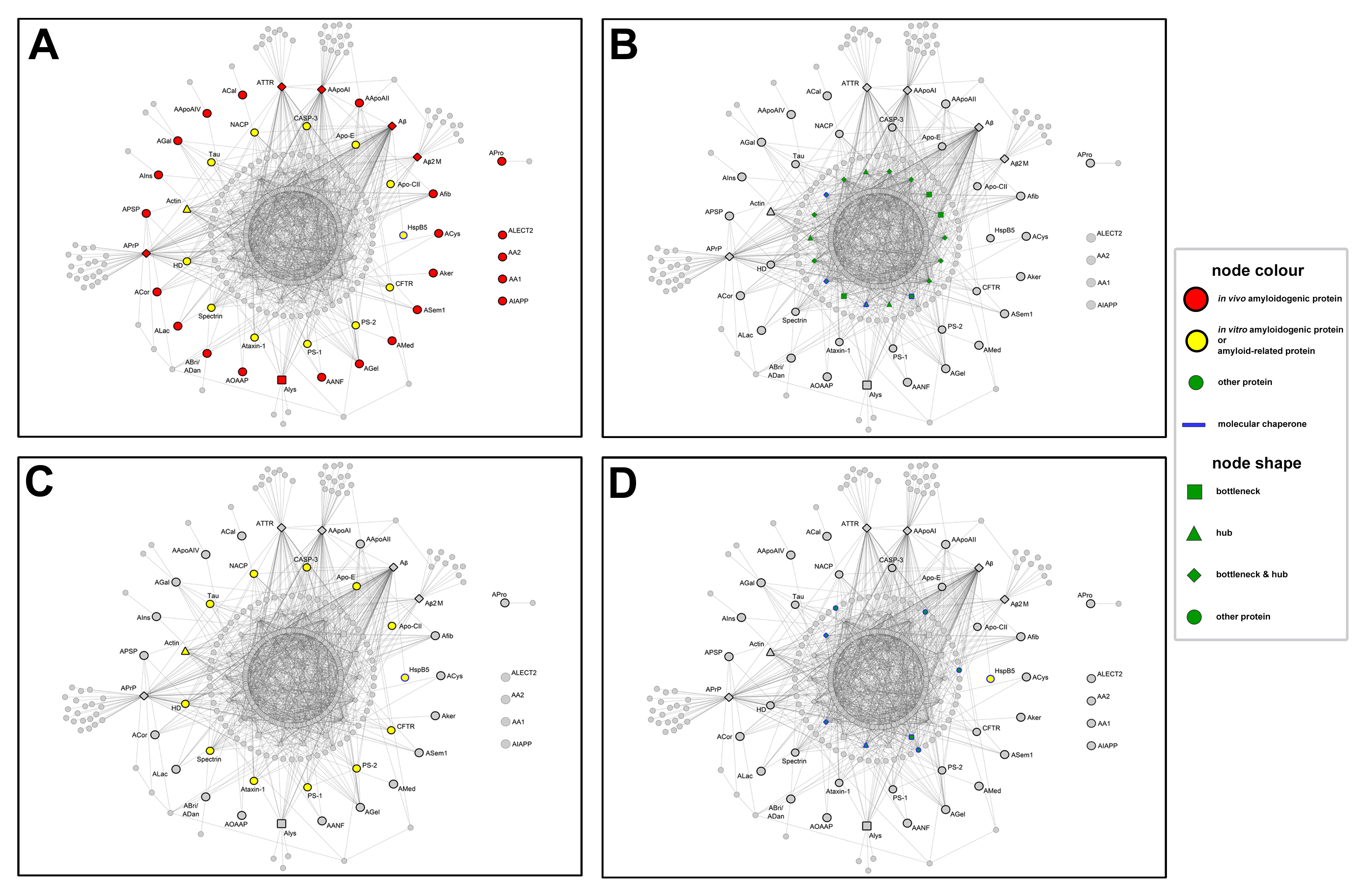

Supplement: S3 Fig — (A) Interactions between in vivo amyloidogenic proteins (red-coloured nodes) and in vitro amyloid forming proteins or proteins related to amyloid fibril formation (yellow-coloured nodes). (B) Yellow-coloured nodes represent in vitro amyloid forming proteins or proteins related to amyloid fibril formation and are a delicate feature of the amyloid interactome. (C) Representation of the key role of non-amyloidogenic hubs and bottlenecks in the amyloid interactome. Triangles are proteins acting as hubs, squares are proteins acting as bottlenecks and diamonds are proteins acting as both. (D) Nodes with blue borders represent proteins characterized as chaperones or co-chaperones (Interactive network available at http://83.212.109.111/amyloid_interactome). (TIF) [file pone.0173163.s003.tif]

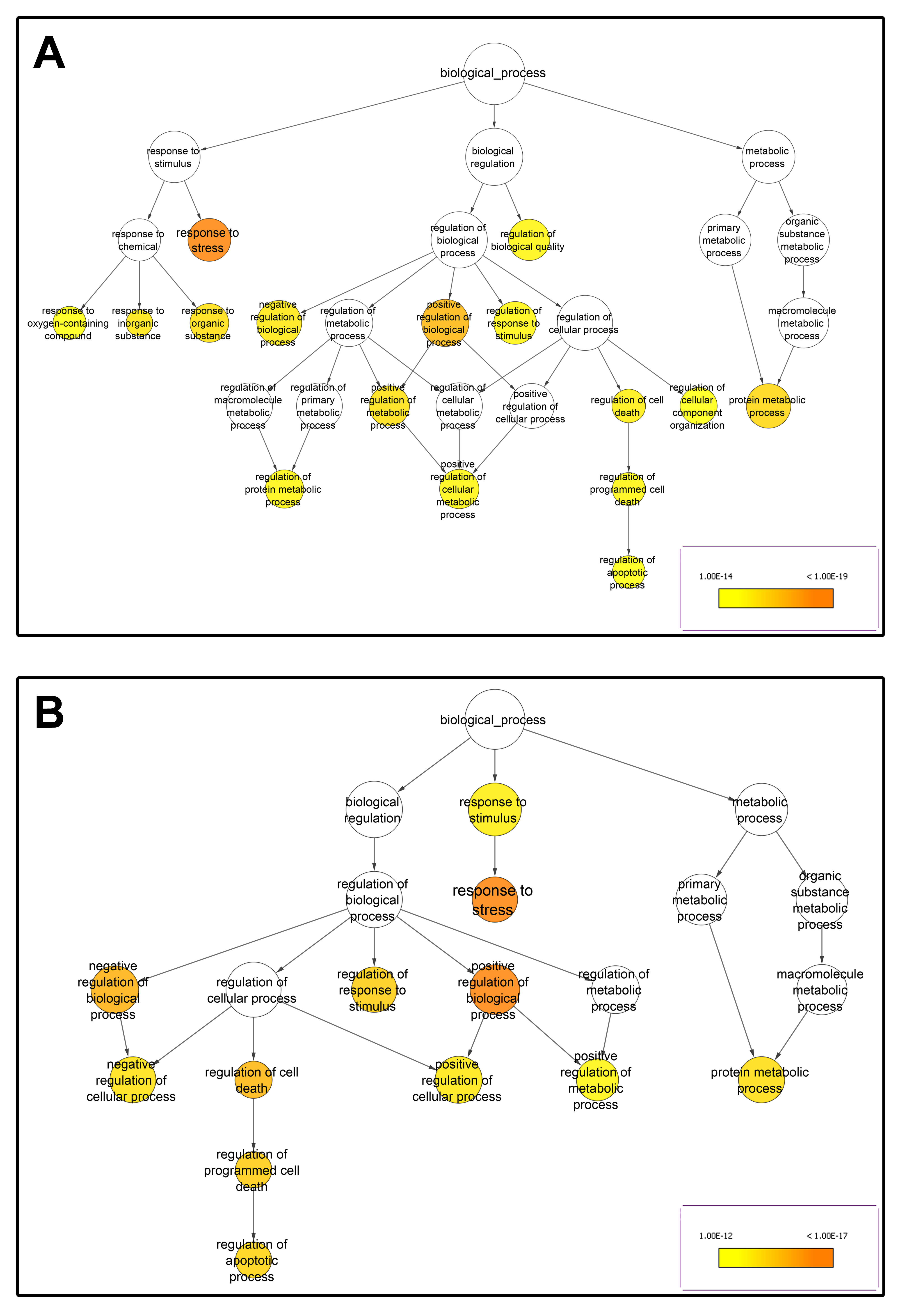

Supplement: S4 Fig — Functionally grouped networks of enriched categories were generated both for the amyloid interactome (A) and cluster 1 (B). GO terms are represented as nodes. The colour gradient of each circle corresponds to the p-value of the associated GO term. White-coloured nodes are not statistically significant nodes, but are parent nodes of statistically significant GO terms. Different node sizes are indicative of varying frequencies of the proteins correlated with each GO term (See S4 and S6 Tables). (TIF) [file pone.0173163.s004.tif]
