# Supplementary material for: The amyloid interactome: Exploring protein aggregation
Source: PLoS One. 2017 Mar 1;12(3):e0173163. doi: 10.1371/journal.pone.0173163 (PMC5383009; doi:10.1371/journal.pone.0173163)
Supplement: S1 Table — This extended list of proteins includes proteins known to self-assemble into typical amyloid fibrils in vivo, along with intracellular inclusions with known biochemical composition, published by Sipe et al. in 2014 [2]. In addition to this, the list includes proteins which form amyloid fibrils in vitro [45], protein segments obtained from AmyLoad [5] and finally, UniProtKB entries [46] of proteins related to amyloid fibril formation. A UniProtKB Accession Number is provided for each protein. The original source library of each protein is tagged with a cross (+). The Digital Object Identifier (DOI) code is provided, when it is available (See Materials and methods). (PDF) [file pone.0173163.s005.pdf]

S1 Table. The non-redundant, detailed catalogue of *in vivo* and *in vitro* amyloidogenic proteins or peptide fragments or proteins related to amyloid fibril formation

Amyloidogenic Protein Name	Uniprot AC	Source Literature / Source Database					Fragment	Digital Object Identifier(DOI)
		**Sipe, et al. (2014) Amyloid** 21(4):221-224	**Harisson, et al. (2007) Rev. Physiol. Biochem. Pharmacol.** 159:1 –77	**Uversky, et al. (1999) Med. Sci. Monit.** 5(5): 1001-1012 & **Med. Sci. Monit.** 5(6): 1238-1254	**AmyLoad Wozniak & Kotulska (2015) Bioinformatics** 31(20):3395-3397	**UniProt UniProt Consortium (2015) Nucl. Acids Res.** 43 (D1): D204-D212		
**4F2 cell-surface antigen heavy chain**	P08195				+		✓	no data
**60 kDa SS-A/Ro ribonucleoprotein**	P10155				+		✓	no data
**Acetylcholinesterase fragment**	P22303		+				✓	10.1021/bi0260334
**Actin, cytoplasmic 1, ACTB, Beta-actin**	P60709	+						no data
**Actin,cytoplasmic 2, ACTG1, Gamma-actin**	P63261	+						no data
**Acylphosphatase-2**	P14621				+		✓	10.1038/nmeth.1432
**alpha beta crystallin (ABC) (Alpha-crystallin B chain)**	P02511				+		✓	10.1126/science.1213151
**Alpha-(1,3)-fucosyltransferase 9**	Q9Y231				+		✓	no data
**Alpha-galactosidase A precursor**	P06280				+		✓	no data
**alpha-synuclein**	P37840	+	+	+			✓	10.1042/bst0300559
**Alstrom syndrome protein 1**	Q8TCU4				+		✓	no data
**Aminopeptidase N**	P15144				+		✓	10.1038/nmeth.1432
**Amphoterin**	P09429		+				✓	10.1021/bi002095n
**Amyloid beta A4 protein**	P05067	+	+	+			✓	10.1074/jbc.274.18.12619
**Androgen receptor protein**	P10275		+	+				10.1007/s00109-004-0530-7
**Apolipoprotein A-I**	P02647	+	+	+			✓	10.1074/jbc.M204801200

**Apolipoprotein A-II**	P02652	+	+				✓	10.1006/geno.2000.6499
**Apolipoprotein A-IV**	P06727	+	+					10.1006/bbrc.2001.5260<br>10.1038/labinvest.3700124
**Apolipoprotein E**	P02649					+		10.1093/jnen/60.4.342<br>10.1212/01.wnl.0000258354.96336.97
**Apoliprotein C-II**	P02655				+		✓	10.1016/j.jmb.2006.12.040
**Aquaporin-2**	P41181				+		✓	10.1038/nmeth.1432
**Ataxin 1**	P54253		+	+				PMID: 9342197
**Ataxin 2**	Q99700		+					10.1016/S0014-4886(03)00287-5
**Ataxin 3**	P54252		+					10.1021/bi0352825
**ATP-binding cassette sub-family A member 12**	Q86UK0				+		✓	no data
**Atrophin-1**	P54259		+					PMID: 11037186
**a-tubulin (Tubulin alpha-1B chain)**	P68363				+		✓	10.1006/bbrc.1996.0211
**Bardet-Biedl syndrome 5 protein**	Q8N3I7				+		✓	10.1038/nmeth.1432
**Beta-2-microglobulin**	P61769	+	+	+			✓	10.1016/S0022-2836(02)01227-5
**Beta-hexosaminidase subunit alpha precursor**	P06865				+		✓	10.1038/nmeth.1432
**Bloom syndrome protein**	P54132				+		✓	10.1038/nmeth.1432
**CA150 (Transcription elongation regulator 1)**	O14776		+				✓	10.1073/pnas.0607815103
**CAAX prenyl protease 1 homolog**	O75844				+		✓	no data
**Calcitonin**	P01258	+	+	+			✓	10.1074/jbc.M206039200
**Caspase-3 precursor (subunit p17)**	P42574				+		✓	10.1038/nmeth.1432
**Coagulation factor XIII B chain**	P05160		+					no data
**Coagulation factor XIII, A chain**	P00488		+					no data
**Collagen alpha-1**	Q9BXS0					+		10.1093/emboj/21.7.1524<br>10.1074/jbc.M403628200
**Corneodesmosin**	Q15517	+						no data
**Cystatin A**	P01040		+					10.1002/prot.20041
**Cystatin B**	P04080		+					10.1016/S0167-4838(01)00295-3

**Cystatin-C**	P01034	+	+	+				10.1002/prot.20633
**Cystic fibrosis transmembrane conductance regulator**	P13569				+		✓	10.1038/nmeth.1432
**Disintegrin and metalloproteinase domain-containing protein 10**	O14672					+		10.1093/hmg/ddp323
**DNA mismatch repair protein Mlh3**	Q9UHC1				+		✓	no data
**Endostatin (Collagen alpha-1(XVIII) chain)**	P39060		+				✓	10.1021/bi048510j
**Ferritin heavy chain**	P02794	+						no data
**Ferritin light chain**	P02792	+						10.1212/01.wnl.0000178224.81169.c2
**Fibrinogen alpha chain**	P02671	+	+					10.1038/ng0393-252
**Galectin 7**	P47929	+						no data
**Gamma-aminobutyric acid receptor subunit beta-1 precursor**	P18505				+		✓	no data
**Gelsolin**	P06396	+	+	+			✓	PMID: 14640038
**Glycophorin A**	P02724		+				✓	10.1021/bi047827g
**G-protein coupled receptor 20**	Q99678				+		✓	no data
**GRB2-associated-binding protein 1**	Q13480				+		✓	no data
**Growth/differentiation factor 3 precursor**	Q9NR23				+		✓	no data
**Guanine nucleotide-binding protein subunit beta-5**	O14775				+		✓	no data
**Histone-lysine N-methyltransferase 2C**	Q8NEZ4				+		✓	no data
**Homeobox protein DLX-3**	O60479				+		✓	no data
**Huntingtin (Polyq expanded)**	P42858	+	+	+				10.1016/S0896-6273(02)00872-3
**Ig gamma-1 chain C region (Immunoglobulin Heavy Chain)**	P01857	+	+					PMID: 2118650
**Ig kappa chain C region (Immunoglobulin Light Chain)**	P01834	+	+	+			✓	10.1074/jbc.M207225200
**Ig kappa chain V-I region BAN**	P04430					+		no data
**Ig kappa chain V-II region TEW**	P01617					+		
**Ig lambda chain V-I region EPS**	P06888					+		no data

**Ig lambda chain V-II region NIG-84**	P04209					+		
**Ig lambda chain V-IV region MOL**	P06889					+		no data
**Ig lambda chain V-VI region AR**	P01721					+		
**Inner nuclear membrane protein Man1**	Q9Y2U8				+		✓	no data
**Insulin**	P01308	+	+					PMID: 6337294<br>10.4103/2230-8210.146879
**Integral membrane protein 2B**	Q9Y287	+	+				✓	10.1042/BST20051111
**Integrin alpha-8 precursor (extracellular domain)**	P53708				+		✓	no data
**Interleukin-31 receptor subunit alpha**	Q8NI17					+		10.1038/ejhg.2009.135
**Islet amyloid polypeptide**	P10997	+	+	+			✓	10.1006/jmbi.1999.3422
**Kerato-epithelin**	Q15582	+	+					PMID: 9799082
**Kv channel-interacting protein 1**	Q9NZI2				+		✓	no data
**Lactadherin**	Q08431	+	+				✓	10.1073/pnas.96.15.8669
**Lactoferrin**	P02788	+	+				✓	10.1021/bi0204746
**Leukocyte cell-derived chemotaxin-2**	O14960	+						no data
**Low-density lipoprotein receptor precursor**	P01130				+		✓	no data
**Lysozyme C**	P61626	+	+	+				10.1002/humu.9393
**Major prion protein**	P04156	+	+	+			✓	PMID: 1438300<br>10.1046/j.1471-4159.2003.01664.x
**NACHT, LRR and PYD domains-containing protein 3**	Q96P20					+		10.1002/art.10509
**NADH-ubiquinone oxidoreductase chain 1**	P03886					+		10.1006/geno.1993.1299
**NADH-ubiquinone oxidoreductase chain 2**	P03891					+		10.1016/S0006-291X(05)80136-6
**Natriuretic peptides A**	P01160	+	+					10.1016/S0006-291X(87)80243-7
**Neuroserpin**	Q99574	+						10.1038/43894
**Nucleophosmin**	P06748				+		✓	10.1096/fj.14-269522
**Odontogenic ameloblast-associated protein**	A1E959	+	+				✓	10.1042/BST20051111

**Oncostatin-M-specific receptor subunit beta**	Q99650				+		10.1016/j.ajhg.2007.09.002
**Origin recognition complex subunit 3**	Q9UBD5			+		✓	no data
**Otoferlin**	Q9HC10			+		✓	no data
**P21-ras (GTPase HRas)**	P01112			+		✓	PMID: 7723021
**p53**	P04637		+				10.1016/S0022-2836(03)00175-X
**Phosphatidylinositol 3-kinase regulatory subunit alpha**	P27986			+		✓	10.1038/nmeth.1432
**Plasminogen activator inhibitor 1 precursor**	P05121			+		✓	10.1038/nmeth.1432
**Polyadenine-binding protein 2**	Q86U42		+				10.1159/000072861
**Polycystin-1 precursor**	P98161			+		✓	10.1038/nmeth.1432
**Polypeptide N-acetylgalactosaminyltransferase 1**	Q10472			+		✓	no data
**POU domain, class 2 transcription factor 1**	P14859			+		✓	10.1038/nmeth.1432
**Presenilin 1**	P49768		+			✓	10.1006/bbrc.1997.6336
**presenilin 2**	P49810		+			✓	10.1006/bbrc.1997.6336
**Prion-like protein doppel**	Q9UKY0				+		no data
**Procarboxypeptidase A2 activation domain**	P48052		+			✓	10.1042/bj20030368
**Prolactin**	P01236	+	+			✓	PMID: 9006323
**Proprotein convertase subtilisin/kexin type 6 precursor**	P29122			+		✓	no data
**Prosaposin receptor GPR37 precursor**	O15354			+		✓	no data
**Prostatic acid phosphatase**	P15309				+	✓	10.1016/j.cell.2007.10.014<br>10.1128/JVI.00268-09
**Protein unc-13 homolog B**	O14795			+		✓	no data
**Protocadherin beta-2 precursor**	Q9Y5E7			+		✓	10.1038/nmeth.1432
**Protocadherin-15 precursor**	Q96QU1			+		✓	no data
**Pulmonary surfactant-associated protein C**	P11686	+	+			✓	10.1056/NEJMra023226
**Putative BPIFA4P protein precursor**	Q86YQ2			+		✓	no data

**Pyrin**	O15553					+		10.1038/sj.ejhg.5200303
**Relaxin receptor 2**	Q8WXD0				+		✓	10.1038/nmeth.1432
**Ribose-5-phosphate isomerase**	P49247				+		✓	no data
**RNA-binding protein FUS**	P35637				+		✓	
**RNA-binding protein Musashi homolog 1**	O43347				+		✓	no data
**Rod cGMP-specific 3', 5'-cyclic phosphodiesterase subunit alpha precursor**	P16499				+		✓	no data
**Semenogelin-1**	P04279	+	+				✓	PMID: 15962837
**Serine/threonine-protein phosphatase 5**	P53041					+	✓	10.1074/jbc.M410775200
**Serum amyloid A-1**	P0DJI8	+	+	+			✓	10.1016/S0006-291X(05)80107-X
**Serum amyloid A-2**	P0DJI9	+	+	+			✓	10.1016/S0006-291X(05)80107-X
**Serum amyloid P-component**	P02743					+		PMID: 4055725
**Shadow of prion protein**	Q5BIV9					+		no data
**SLIT and NTRK-like protein 5 precursor**	O94991				+		✓	no data
**Sortilin-related receptor**	Q92673					+		10.1126/scitranslmed.3007747<br>10.1038/mp.2012.15
**Spectrin SH3 (Spectrin alpha chain, non-erythrocytic 1)**	Q13813				+		✓	10.1006/jmbi.1996.0042
**Splicing factor 3A subunit 2**	Q15428				+		✓	no data
**Superoxide dismutase 1**	P00441		+					10.1038/362059a0
**TAR DNA-binding protein 43**	Q13148				+		✓	
**Taste receptor type 2 member 5**	Q9NYW4				+		✓	10.1038/nmeth.1432
**TATA-box binding protein**	P20226		+					10.1093/hmg/10.14.1441
**Tau**	P10636	+	+	+			✓	10.1074/jbc.M402379200
**Titin**	Q8WZ42				+		✓	10.1038/nmeth.1432
**Transportin-1**	Q92973				+		✓	10.1038/nmeth.1432

**Transthyretin**	P02766	+	+	+				10.1007/PL00000791
**Tumor necrosis factor receptor superfamily member 1A**	P19438					+		10.1086/321976
**Tumor-associated calcium signal transducer 2**	P09758					+		10.1038/7759
**Tyrosine-protein kinase Mer precursor**	Q12866				+		✓	no data
**Tyrosine-protein kinase transmembrane receptor ROR2 precursor**	Q01974				+		✓	no data
**Unconventional myosin-XV**	Q9UKN7				+		✓	10.1038/nmeth.1432
**YLP motif-containing protein 1**	P49750				+		✓	10.1038/nmeth.1432
**α1 Anti-trypsin C-terminal peptides**	P01009		+				✓	PMID: 7576238
**α1A voltage-dependent calcium channel subunit**	O00555		+					10.1159/000072849

AC, Accession;
***Protein Names were extracted from UniProt**
