# Supplementary material for: The amyloid interactome: Exploring protein aggregation
Source: PLoS One. 2017 Mar 1;12(3):e0173163. doi: 10.1371/journal.pone.0173163 (PMC5383009; doi:10.1371/journal.pone.0173163)
Supplement: S2 Table — The 20 proteins with the highest node degrees are considered as hubs in the amyloid interactome. 6 of these proteins belong to the dataset of the amyloidogenic proteins, described in S1 Table, whilst the rest of the hubs exhibit numerous functions, acting mainly as chaperones, signal transducers or structural constituent of the cell (See Results and discussion). (PDF) [file pone.0173163.s006.pdf]

S2 Table. The top 20 hubs of the amyloid interactome.

Protein names*	Degree
**Amyloid beta A4 protein**	**84**
**Apolipoprotein A-I**	**61**
**Major prion protein**	**57**
Transcription factor AP-1	53
**Transthyretin**	**47**
Epidermal growth factor receptor	40
14-3-3 protein zeta/delta	40
Growth factor receptor-bound protein 2	35
Gamma-aminobutyric acid receptor-associated protein-like 1	33
Serum albumin	33
Gamma-aminobutyric acid receptor-associated protein-like 2	33
NF-kappa-B essential modulator	29
Fibronectin	26
Vimentin	25
Heat shock cognate 71 kDa protein	24
Beta-arrestin-1	23
Myosin-9	23
Hsp90 co-chaperone Cdc37	23
**Beta-2-microglobulin**	**23**
Actin, cytoplasmic 1	22

Amyloidogenic proteins are highlighted in bold.
*Protein nomenclature follows the most cited abbreviations in literature.
