# Supplementary material for: The amyloid interactome: Exploring protein aggregation
Source: PLoS One. 2017 Mar 1;12(3):e0173163. doi: 10.1371/journal.pone.0173163 (PMC5383009; doi:10.1371/journal.pone.0173163)
Supplement: S3 Table — The 20 proteins with the highest betweeness centralities are considered bottlenecks in the amyloid interactome. 6 of these proteins belong to the dataset of the amyloidogenic proteins, described in S1 Table. 15 bottlenecks exhibit high node degree values and are also considered as hubs in this network (S2 Table). (PDF) [file pone.0173163.s007.pdf]

S3 Table. The top 20 bottlenecks of the amyloid interactome.

Protein names	Betweenness Centrality
**Amyloid beta A4 protein**	**0.26989904**
**Apolipoprotein A-I**	**0.18986417**
**Major prion protein**	**0.18065884**
**Transthyretin**	**0.10686298**
Transcription factor AP-1	0.08605723
**Beta-2-microglobulin**	**0.07022626**
Serum albumin	0.0484804
Large proline-rich protein BAG6	0.0446524
Growth factor receptor-bound protein 2	0.04446241
Epidermal growth factor receptor	0.04438062
Fibronectin	0.03676366
**Lysozyme C**	**0.0286479**
NF-kappa-B essential modulator	0.02801342
14-3-3 protein zeta/delta	0.02671415
Hsp90 co-chaperone Cdc37	0.02489765
Gamma-aminobutyric acid receptor-associated protein-like 2	0.02366865
Peptidyl-prolyl cis-trans isomerase NIMA-interacting 1	0.02137914
Myc proto-oncogene protein	0.02129564
Gamma-aminobutyric acid receptor-associated protein-like 1	0.02022756
Mitogen-activated protein kinase 6	0.01801841

Amyloidogenic proteins are highlighted in bold.
*Protein nomenclature follows the most cited abbreviations in literature.
