# Supplementary material for: The amyloid interactome: Exploring protein aggregation
Source: PLoS One. 2017 Mar 1;12(3):e0173163. doi: 10.1371/journal.pone.0173163 (PMC5383009; doi:10.1371/journal.pone.0173163)
Supplement: S4 Table — Proteins of the entire amyloid interactome were subjected to a GO term enrichment analysis using BiNGO [56]. The UniProtKB ACs of proteins that are characterized by overrepresented GO terms in the entire amyloid interactome are given in this table, along with their number and their frequency in the network. The adjusted p-value suggests the importance of these GO terms in the proteins of the amyloid interactome. (PDF) [file pone.0173163.s008.pdf]

S4 Table. Amyloid interactome GO term enrichment. p<10E-14 was set as a threshold for Biological Process and Cellular Component whereas p<10E-8 was set as threshold for Molecular Function

GO ID	GO term	Adjusted p-value	No. of proteins	freq (%)	Proteins (Uniprot ACs)
					**Biological Process**
**6950**	response to stress	1.42E-21	143	44.0	Q9H0E2 P07900 P10636 P49662 P29372 Q86VQ3 P62993 P07196 Q92731 P01876 P60059 P49810 P01871 Q16594 P09936 O43681 P78536 Q92561 P02679 P01106 P46379 P00533 P06396 P05067 P42574 O14798 P02675 P49407 P01100 P49768 P52333 P08779 P29353 P32121 P10451 P30101 P14136 Q7Z333 P02747 P09471 P21860 P02745 P01137 P01258 Q9H4B4 P63104 P55854 P01891 O00459 P23142 O43542 Q07325 Q9H0R8 O00213 P35228 Q9C000 P21359 P19838 Q16543 P60520 P07585 Q96JB5 P25713 Q03518 Q8WYR1 P10909 P04279 O95477 P01160 P36544 O94782 P35579 P02649 Q9Y6K9 Q99683 P01834 P02647 P04040 P02768 Q30201 P61626 P69905 Q14011 P01034 O75509 P07437 P01275 Q8IY92 P05412 P60709 Q15109 P22392 O43889 P37231 O14879 P11142 P00738 Q00987 P01308 P00734 P54829 P23634 P02511 P08138 P0DJI8 P02751 P0DJI9 P27797 P01023 P02671 Q5MNZ9 Q7L5D6 P49840 Q8WTV0 P58753 P31751 Q08345 Q9UQF2 P60033 P01857 P61764 P02788 P02786 P04264 P61769 O14920 P60953 Q92876 Q8N6L0 O75955 Q96FW1 P06727 P37840 P00519 A1E959 O00141 P02656 Q9UMX0 P40337 P61812 P02652 P04156 P21796
**48518**	positive regulation of biological process	9.84E-18	179	55.1	Q9H0E2 Q15654 P07900 P10636 P13984 P62993 Q9NX24 P07196 Q92731 P01876 P49810 P01236 P01871 Q16594 P62633 Q13642 P78536 Q13526 O95163 Q07343 Q9P2S5 Q9BT40 Q08431 P19338 P42858 P02679 P01106 P46379 P00533 P06396 Q9BRP8 P05067 Q99759 P42574 P02675 P49407 P01100 P49768 P52333 P29353 P62937 P32121 P13569 P10451 P30101 P14136 Q7Z333 P02747 P21860 P02745 P25788 P03372 P01137 P01258 Q9H4B4 P63104 Q15047 P61964 P49757 O00459 O95741 P23142 P20393 O43542 Q07325 P68133 Q08379 O00213 P35228 Q9NP59 Q9C000 Q93074 P21359 P19838 P08670 Q15572 P61457 P07585 Q96JB5 P25713 Q03518 P41743 Q8WYR1 P10909 P04279 Q13951 O95477 Q9UL18 P01160 P36544 Q8TD08 P35579 Q99689 P02649 Q9Y6K9 Q99683 P01834 P02647 P04040 P02768 Q30201 P78352 P69905 Q9UJW9 Q13043 Q14011 O75509 P01275 Q8IY92 P05412

					P07711 Q68CZ2 Q9P1U0 P60709 Q15109 P22392 O43889 Q9UBX2 P37231 P11142 P00738 Q00987 P01308 P00734 P54829 P23634 P02753 P08138 Q15113 P0DJI8 P02751 P27797 P46459 Q05193 P02671 Q92913 P49840 Q8WTV0 P58753 Q96L34 P31751 Q9NW64 Q8IUH5 Q9UQF2 Q92870 P60033 P01857 P01614 P61764 P02788 Q96RG2 P02787 P02786 P04264 P61769 O14920 Q9BSE2 P60953 Q92876 Q7Z5J4 O75955 Q13625 P06727 O75674 Q38SD2 P37840 P00519 A1E959 O00141 Q9NPJ6 P02656 P02655 Q9UMX0 P02654 P40337 Q15653 P61812 Q01814 P02652
**19538**	protein metabolic process	1.92E-16	156	48.0	P22061 P07900 P10997 P49662 P11686 P62993 Q9NX24 P07196 P01876 P49810 P01236 O75829 P01871 Q16594 P09936 P78536 Q13526 O95163 Q8NB14 Q08431 P68104 Q9Y2H9 P02679 P01106 P46379 P00533 P06396 P05067 Q99759 P42574 Q9UHP3 P02675 P49407 P49768 P52333 O75529 Q13813 Q9Y287 P29353 P62937 P32121 P56817 P30101 Q7Z569 Q9Y4E8 P04180 Q7Z333 P02747 P21860 P02745 P25788 P01137 P01258 Q9H4B4 P27448 Q15047 P61964 P55854 Q16659 O43781 P23142 P07339 P55212 P20393 Q08379 O00213 P35228 Q9C000 Q93074 P21359 P19838 Q16543 P62945 P07585 Q96JB5 P25713 P41743 P10909 P04279 Q13951 O95477 P01160 Q8TD08 O94782 P35579 P02649 Q9Y6K9 Q99683 P01834 P02647 P04040 P02768 Q15582 P02766 P78352 P61626 Q13043 P01034 Q12873 Q92529 P07711 Q15109 P22392 O95084 P37231 Q00987 Q96EN8 P01308 P00734 P54829 P02511 P08138 Q15113 P0DJI8 P02751 P27797 P01023 P02671 Q92913 Q5MNZ9 Q7L5D6 P49840 O75688 Q8WTV0 Q96L34 P31751 Q08345 Q8IUH5 Q9UQF2 P01857 P01614 P47897 P02788 Q96RG2 P04264 P61769 O14920 P60953 Q92876 Q11203 Q96FW1 P06727 O75674 Q38SD2 Q86Y79 P37840 P00519 A1E959 O00141 P02656 P02655 Q9UMX0 P02654 P40337 P61812 P02652
**10033**	response to organic substance	4.57E-16	113	34.8	P07900 P10636 P49662 P13984 P62993 P07196 Q92731 P60059 P01236 P02686 Q16594 O43681 P78536 P09417 Q07343 Q9BT40 Q08431 P19338 P68104 P09543 P01106 P46379 P00533 P06396 P05067 P42574 O14798 P02675 P01100 P52333 P29353 P32121 P13569 P10451 Q9Y4E8 P04180 Q7Z333 P09471 P25788 P03372 P01137 P01258 Q15047 P01891 O00459 P20393 O43542 Q07325 P68133 P35228 Q9C000 Q93074 P19838 P07585 Q96JB5 P25713 P41743 P10909 O95477 Q9UL18 P01160 P50053 P36544 Q8TD08 Q99689 P02647 P04040 P02768 Q30201 O75509 P01275 P05412 P07711 Q8NEC5 Q15109 O43889 P37231 O14879 P11142 Q00987 P01308 P54829 P23634 P02753 P02511 P08138 P05783 P02751 P27797 P02671 Q5MNZ9 Q7L5D6 P49840 Q8WTV0 P58753 P31751 P60033 Q9BRK5 P02786 P61769 O14920 O75955 Q96FW1 P06727 P37840 P00519 O00141 Q9NPJ6 P02656 Q9UMX0 Q15653 P61812 P02652

**9893**	positive regulation of metabolic process	5.05E-16	124	38.2	Q9H0E2 P07900 P13984 P62993 Q92731 P01876 Q16594 P62633 P78536 Q13526 Q9BT40 P19338 P02679 P01106 P46379 P00533 P06396 Q9BRP8 P05067 Q99759 P42574 P02675 P49407 P01100 P49768 P29353 P32121 Q7Z333 P21860 P25788 P03372 P01137 P01258 Q9H4B4 P61964 O00459 P23142 P20393 Q07325 P68133 Q08379 O00213 P35228 Q9NP59 Q9C000 Q93074 P21359 P19838 P08670 Q15572 P61457 P07585 Q96JB5 P25713 P41743 Q8WYR1 P10909 P04279 Q13951 O95477 Q9UL18 P36544 Q8TD08 P35579 P02649 Q9Y6K9 Q99683 P02647 P04040 Q30201 Q9UJW9 Q13043 Q14011 P01275 Q8IY92 P05412 Q9P1U0 P60709 Q15109 P22392 O43889 Q9UBX2 P37231 P11142 Q00987 P01308 P00734 P54829 P23634 P08138 Q15113 P0DJI8 P02751 P27797 P46459 P02671 Q92913 P49840 Q8WTV0 P58753 P31751 Q9NW64 P60033 P02788 Q96RG2 O14920 P60953 Q7Z5J4 O75955 P06727 O75674 Q38SD2 P37840 P00519 A1E959 Q9NPJ6 P02656 P02655 Q9UMX0 P02654 P40337 Q15653 P61812 P02652
**10035**	response to inorganic substance	8.76E-16	45	13.8	P00450 P02671 P29353 P50053 P04180 P07196 Q7Z333 Q99683 P09471 Q92731 P04040 P02768 Q30201 P02745 P02787 P02786 P69905 P61769 P02686 P05412 O95741 P09417 Q15109 P06727 P68133 P37840 O00213 Q9NP59 P00738 P00519 Q00987 P19838 P02679 P23634 P25713 P06396 P02511 P05067 P42574 P02675 P02751 P04156 P27797 P10909 P01100
**48519**	negative regulation of biological process	1.08E-15	159	48.9	Q96DZ9 Q15654 P10997 P10636 Q9Y3Q8 P62993 P07196 Q92731 P49810 O75829 P02686 Q16594 P09936 Q13642 P78536 Q13526 Q07343 Q9P2S5 Q9BT40 P19338 P42858 P02679 P01106 P46379 P00533 P06396 P05067 P42574 Q9UHP3 P02675 P49407 P49768 P52333 Q8N205 P08779 Q13813 Q9Y287 P32121 P10451 Q7Z569 P14136 Q7Z333 P02747 P09471 P21860 P25788 P03372 P01137 P01258 Q9H4B4 P63104 P49757 P23142 P20393 Q08379 O00213 P35228 Q9NP59 Q9NRR5 Q93074 P21359 P19838 P08670 P60520 P07585 Q96JB5 P25713 Q03518 P41743 P10909 P04279 O95477 Q9UL18 P01160 P54253 P36544 Q8TD08 P35579 Q99689 P02649 Q9Y6K9 P02647 P04040 P02768 Q30201 Q15582 P78352 Q9UJW9 Q13043 Q14011 P01034 O75509 Q12873 P07437 P01275 P05412 Q15109 P22392 O43889 P50502 Q9UBX2 P37231 O14879 P11142 P00738 Q00987 P01308 P00734 P54829 P46695 P23634 P02753 P02511 P08138 P05783 P0DJI8 P02751 P27797 P01023 Q05193 P02671 Q92913 Q6UXB4 P49840 O75688 P58753 P31751 Q08345 Q9P2H0 Q9UQF2 Q92870 P61764 P02788 Q96RG2 P04264 P61769 O14920 P60953 Q7Z5J4 Q9HCC9 Q13625 Q96FW1 P06727 O75674 Q38SD2 P37840 P00519 O00141 P02656 P02655 Q9UMX0 P02654 P40337 Q15653 P61812 Q14684 P02652 P04156 P21796
**10941**	regulation of cell	1.75E-15	80	24.6	P49662 P07196 P02649 Q9Y6K9 Q99683 Q92731 P04040 P02768 P49810 P69905

	death				Q13043 Q16594 O75509 P01275 P05412 P78536 Q13526 Q15109 P22392 O43889 P19338 P37231 O14879 P00738 Q00987 P42858 P01308 P54829 P46695 P02679 P01106 P46379 P00533 P06396 P02511 P42574 P08138 P05783 O14798 P02675 P49407 P02751 P27797 P49768 P52333 P02671 P49840 P32121 Q96L34 P31751 P30101 Q9UQF2 Q92870 Q7Z333 P21860 P61764 P02788 P03372 P01137 Q9H4B4 P63104 O14920 P60953 P55212 Q13625 P37840 O00213 Q9NP59 Q9C000 P21359 P00519 P19838 O00141 P25713 Q9UMX0 P40337 P61812 P41743 P04156 P10909
**1901700**	response to oxygen-containing compound	2.60E-15	77	23.7	O95477 P01160 P49662 P50053 Q8TD08 P62993 P07196 P02649 Q99683 Q92731 P04040 P01236 P69905 P02686 O75509 P01275 P05412 P78536 Q8NEC5 P09417 Q15109 Q07343 Q9BT40 P19338 P37231 P00738 Q00987 P01308 P54829 P09543 P23634 P00533 P06396 P02753 P02511 P05067 P42574 P08138 O14798 P02751 P27797 P01100 P52333 P02671 P49840 P29353 P13569 Q8WTV0 P58753 P31751 P10451 Q7Z333 P09471 Q9BRK5 P03372 P01137 Q9H4B4 P02786 P61769 O14920 Q15047 O00459 P20393 P06727 Q07325 P37840 P35228 Q9C000 P00519 P19838 P02656 P07585 P25713 Q15653 P61812 P41743 P02652
**31325**	positive regulation of cellular metabolic process	3.63E-15	117	36.0	Q9H0E2 P07900 P62993 Q92731 Q16594 P09936 P78536 Q13526 O95163 Q9BT40 P68104 P42858 P02679 P01106 P46379 P00533 P06396 Q9BRP8 P05067 Q99759 P42574 Q9UHP3 P02675 P49407 P49768 P52333 Q9Y287 P29353 P32121 P14136 P21860 P25788 P03372 P01137 P01258 Q9H4B4 P23142 O43542 Q96A00 Q08379 O00213 P35228 Q9C000 Q9NRR5 P21359 P19838 Q16543 P60520 P07585 Q96JB5 P25713 Q9BT17 Q8WYR1 P10909 P04279 Q9UL18 P36544 Q8TD08 P35579 P02649 Q9Y6K9 Q99683 P02647 Q30201 Q13043 Q14011 P01034 P01275 P05412 Q15109 P50502 P37231 Q00987 P01308 P00734 P54829 P23634 P02511 P08138 Q15113 P0DJI8 P02751 P27797 P46459 P01023 P02671 Q92913 P49840 P58753 P31751 Q9UQF2 P60033 P02788 Q96RG2 O14920 P60953 O75955 Q9HCC9 Q96FW1 P06727 O75674 Q38SD2 P37840 P00519 A1E959 Q9UMX0 P61812 Q14684 P02652 P04156
**48583**	regulation of response to stimulus	3.63E-15	142	43.7	P29016 Q15654 P07900 P49662 P62993 Q92731 P01876 P01236 P01871 Q16594 Q53QZ3 P09936 P78536 Q13526 Q07343 Q9BT40 P42858 Q92561 P02679 P01106 P46379 P00533 P06396 P05067 Q99759 P42574 Q9UHP3 P02675 P49407 P01100 P49768 P52333 P29353 P32121 P13569 Q9Y6Q5 P10451 P30101 Q7Z569 Q9UQB3 P02747 P21860 P02745 P25788 P03372 P01137 Q9H4B4 P63104 P49639 P06126 P49757 P01891 O00459 P23142 P20393 Q07325 O00213 P35228 Q9C000 Q9NRR5 Q93074 P21359 P19838 Q96QH2 Q16543 P07585 Q96JB5 P25713 Q03518 Q8WYR1 P10909 O95477 P36544 O94782 Q99689 P02649 Q9Y6K9 Q99683 P01834 P02647

					P04040 P02768 Q30201 P78352 Q13043 O75509 Q12873 P01275 P05412 P07711 P60709 Q15109 O43889 P37231 P11142 P00738 Q00987 P01308 P00734 P54829 P46695 P23634 P02511 P08138 P0DJI8 P02751 P27797 P01023 Q05193 P02671 Q6UXB4 P49840 O75688 P58753 P31751 Q8IUH5 Q96Q45 Q9UQF2 P60033 P01857 P01614 P02788 P04264 P61769 O14920 P60953 Q92876 O75955 Q9HCC9 Q13625 Q96FW1 Q38SD2 P37840 P55899 P00519 P02656 Q9UMX0 Q15653 P61812 P02652 P04156 P21796
**65008**	regulation of biological quality	3.63E-15	131	40.3	Q9H0E2 P07900 P13984 P62993 Q92731 Q16594 P62633 P78536 Q13526 Q9BT40 P19338 P02679 P01106 P00533 P06396 Q9BRP8 P05067 Q99759 P42574 P02675 P49407 P01100 P49768 P29353 P32121 Q7Z333 P21860 P25788 P03372 P01137 P01258 Q9H4B4 P61964 O00459 P23142 P20393 Q07325 Q08379 O00213 P35228 Q9NP59 Q9C000 Q93074 P21359 P19838 P61457 P07585 Q96JB5 P25713 P41743 Q8WYR1 P10909 P04279 Q13951 O95477 Q9UL18 P36544 Q8TD08 P35579 P02649 Q9Y6K9 Q99683 P02647 P04040 Q30201 Q9UJW9 Q13043 Q14011 P01275 Q8IY92 P05412 Q15109 P22392 O43889 Q9UBX2 P37231 P11142 Q00987 P01308 P00734 P54829 P23634 P08138 Q15113 P0DJI8 P02751 P27797 P46459 P02671 Q92913 P49840 Q8WTV0 P58753 P31751 Q9NW64 P60033 P02788 Q96RG2 O14920 P60953 Q7Z5J4 O75955 P06727 O75674 Q38SD2 P37840 P00519 A1E959 Q9NPJ6 P02656 P02655 Q9UMX0 P02654 P40337 Q15653 P61812 P02652
**51246**	regulation of protein metabolic process	3.63E-15	110	33.8	Q15654 P07900 P10997 P10636 Q86VQ3 P62993 Q9NX24 P07196 Q92731 P01876 P01236 Q16594 Q53QZ3 Q13642 P78536 Q13526 Q07343 Q9BT40 P42858 P02679 P09543 P01106 P46379 P06396 P05067 P42574 P02675 P49407 P49768 P52333 P00450 P08779 Q13813 P29353 P32121 P13569 P10451 P30101 P14136 P04180 P25788 P03372 P01137 P01258 P63104 O00459 O43781 P23142 P20393 O43542 Q07325 P35228 Q9NP59 P21359 P19838 Q16543 P07585 P25713 Q03518 Q8WYR1 P10909 O95477 P01160 P36544 Q8TD08 P35579 Q02410 P02649 P01834 P02647 P02768 Q30201 P02766 P78352 P61626 Q13043 Q14011 P01275 Q8IY92 P05412 Q8NEC5 P60709 Q15109 P37231 P11142 Q00987 P01308 P00734 P54829 P23634 P02753 P08138 P0DJI8 P02751 P27797 P01023 P02671 Q92913 O43570 Q7L5D6 P49840 Q8WTV0 P58753 Q08345 P60033 P61764 P02788 Q96RG2 P02787 P02786 P04264 P61769 P07498 Q9BSE2 P60953 Q92876 Q13625 P06727 Q38SD2 P37840 P00519 O00141 P02656 P02655 P40337 Q15653 P61812 Q01814 P02652 P04156 Q96NN9
**43067**	regulation of programmed cell	4.59E-15	76	23.4	P49662 P07196 P02649 Q99683 Q92731 P04040 P02768 P49810 Q13043 Q16594 O75509 P01275 P05412 P78536 Q13526 Q15109 P22392 O43889 P19338 P37231

	death				O14879 Q00987 P42858 P01308 P46695 P02679 P01106 P46379 P00533 P06396 P02511 P42574 P08138 P05783 O14798 P02675 P49407 P02751 P27797 P49768 P52333 P02671 P49840 P32121 Q96L34 P31751 P30101 Q9UQF2 Q92870 Q7Z333 P21860 P61764 P02788 P03372 P01137 Q9H4B4 P63104 O14920 P60953 P55212 Q13625 P37840 O00213 Q9NP59 Q9C000 P21359 P00519 P19838 O00141 P25713 Q9UMX0 P40337 P61812 P41743 P04156 P10909
**42981**	regulation of apoptotic process	9.51E-15	75	23.1	P49662 P07196 P02649 Q99683 Q92731 P04040 P02768 P49810 Q13043 Q16594 O75509 P01275 P05412 P78536 Q13526 Q15109 P22392 O43889 P19338 P37231 O14879 Q00987 P42858 P01308 P46695 P02679 P01106 P46379 P00533 P06396 P02511 P42574 P08138 P05783 O14798 P02675 P49407 P02751 P27797 P49768 P52333 P02671 P49840 P32121 P31751 P30101 Q9UQF2 Q92870 Q7Z333 P21860 P61764 P02788 P03372 P01137 Q9H4B4 P63104 O14920 P60953 P55212 Q13625 P37840 O00213 Q9NP59 Q9C000 P21359 P00519 P19838 O00141 P25713 Q9UMX0 P40337 P61812 P41743 P04156 P10909
**51128**	regulation of cellular component organization	9.91E-15	102	31.4	P07900 P10636 P13984 Q8NI77 P62993 P07196 Q92731 P02686 Q16594 Q53QZ3 Q13642 P78536 Q13526 Q9P2S5 Q9BT40 Q08431 P42858 P02679 P01106 P00533 P06396 P02675 P49407 P49768 Q8N205 Q13813 P32121 P10451 P14136 P04180 Q7Z333 P01137 P63104 Q15047 P49757 O95741 P23142 O43542 Q07325 Q08379 O00213 Q9NRR5 P08670 P07585 P25713 Q9BT17 P41743 P10909 P01160 Q8TD08 P35579 Q99689 P02649 P02647 Q30201 P78352 Q9UJW9 P01034 Q12873 P07437 P01275 Q8IY92 P05412 Q15109 P37231 P11142 P01308 P00734 P54829 Q66K74 P02511 P08138 P02751 P27797 P02671 Q92913 P49840 Q8WTV0 P31751 Q08345 Q9P2H0 Q92870 P61764 P02787 P61769 O14920 P60953 Q92876 O75955 Q13625 Q96FW1 O75674 P37840 P00519 A1E959 O00141 P02656 P02655 P02654 P61812 P02652 P21796
**Molecular Function**					
**5515**	protein binding	1.38E-26	293	89.3	Q9H0E2 Q15654 P11686 P13984 Q9NX24 P07196 Q9ULW3 Q92731 P60059 P49810 P01871 P09936 O43681 Q07343 Q08431 P68104 P42858 Q9Y2H9 P00533 O14798 Q9UHP3 Q9BSJ6 P52333 P00450 Q8N205 Q13813 Q9Y287 P62937 P32121 P56817 P62380 Q9Y6Q5 Q9H0S4 P10451 P14136 Q9H9D4 Q9UQB3 P09471 P02745 P03372 P63104 P27448 P61964 P55854 Q16659 P01891 P31942 P20393 O43542 Q07325 P68133 Q9H0R8 Q9NP59 Q9C000 P21359 P19838 Q15572 Q9H0A6 Q96QH2 Q16543 P60520 P25713 Q9NPF8 Q03518 P41743 Q8WYR1 P10909 P04279 Q13951

					O95477 Q8IZU0 Q9UL18 P54253 P36544 Q6MZP7 P35579 Q9H081 Q02410 P02649 Q9Y6K9 Q9BWG6 P02647 P04040 P02768 Q15582 P02766 P78352 Q13043 Q14011 Q12873 P07437 Q92529 P60709 Q15109 P22392 O43889 O14879 P11142 P01308 P54829 P48634 P46695 P04053 Q66K74 P02753 P02511 Q15113 P0DJI8 P02751 Q99714 P46459 P02671 Q6UXB4 P49840 O75688 Q5VU43 P58753 Q96L34 P31751 Q8IUH5 Q92870 P60033 Q9BRK5 P47897 P02788 Q14151 P02787 P02786 P04264 P60953 Q92876 Q8N6L0 Q13625 Q96GY3 Q96FW1 O75674 Q38SD2 Q86Y79 P37840 P18669 O00141 P02656 P02655 O95704 Q9UMX0 P40337 P61812 P02652 P04156 Q9BRJ6 P29016 P22061 Q96DZ9 O96006 P07900 P10997 P10636 Q6UXH1 P29372 Q9Y3Q8 Q8NI77 P62993 P01236 Q15388 P02686 Q16594 P62633 Q13642 P78536 P09417 Q13526 O95163 Q9BT40 P19338 P02679 P01106 P46379 P06396 Q9BRP8 P05067 Q99759 P42574 P02675 P49407 O60293 P01100 P49768 O75529 Q9BQN1 P08779 P29353 P13569 P30101 Q7Z569 Q9Y4E8 P04180 Q7Z333 P21860 O14960 P25788 P01137 P01258 Q9H4B4 P49639 Q15047 P06126 P49757 O00459 O95741 P23142 P07339 P55212 Q08379 O00213 P35228 Q9NRR5 Q93074 P08670 Q01850 P61457 P07585 Q96JB5 P01160 P50053 Q9P013 Q8TD08 O94782 Q99689 Q08E93 Q99683 P01834 Q30201 Q96A73 P61626 P69905 Q969X6 Q9UJW9 P01034 O75509 P01275 Q8IY92 P05412 P07711 Q8NEC5 Q68CZ2 P50502 Q9UBX2 Q96IW7 P37231 P00738 Q00987 Q96EN8 Q92484 P00734 P23634 P08138 P05783 P27797 P01023 Q05193 Q92913 Q5MNZ9 Q7L5D6 Q15517 Q8WTV0 Q8TAI1 Q08345 Q9UIF8 Q9NW64 Q58DX5 Q9P2H0 Q9UQF2 P01857 P61764 Q96RG2 P61769 O14920 P07498 Q9BSE2 Q7Z5J4 O75955 Q9HCC9 P06727 P55899 Q8TAG9 P00519 Q969D9 A1E959 Q9NPJ6 Q71U36 Q15653 Q01814 P21796
**42802**	identical protein binding	1.36E-18	76	23.2	P07900 P10997 P54253 P36544 P35579 P62993 P07196 P02649 Q9Y6K9 Q99683 P02647 P04040 P02768 P02766 P61626 Q13043 P01034 P01275 P05412 P09417 P60709 Q15109 P50502 P19338 P37231 O14879 Q00987 P42858 P01308 P00533 Q66K74 P02511 P05067 P02751 Q99714 Q05193 Q15517 Q8WTV0 P58753 Q7Z569 Q8IUH5 Q9Y4E8 Q9UQF2 Q9H9D4 Q7Z333 P21860 O14960 Q9BRK5 P61764 Q14151 P03372 P01137 P01258 P63104 P02786 P61769 O14920 P60953 Q8N6L0 P23142 P55212 Q13625 P06727 Q38SD2 P37840 P35228 Q9NRR5 P19838 P08670 P61457 P02655 Q9UMX0 Q03518 P61812 P02652 P04156
**19899**	enzyme binding	3.09E-13	83	25.3	Q9H0E2 Q15654 O95477 P07900 Q9UL18 P10636 Q99689 P62993 P07196 Q9Y6K9 Q99683 Q9BWG6 Q92731 P02647 P04040 P02768 P78352 P02686 Q16594 P01034 P07437 P05412 P09936 Q92529 P60709 Q13526 P37231 P11142 Q00987 P68104 P01308 P54829 P46379 P23634 P00533 P05067 P42574 P08138 Q9UHP3 P49407

					P02751 P27797 P46459 P01023 P52333 Q05193 Q9BQN1 P49840 P29353 P32121 P13569 Q5VU43 P56817 P58753 P31751 P14136 Q9UQF2 P61764 P03372 P01137 P63104 O14920 P60953 Q16659 O00459 O75955 Q96FW1 O75674 Q9H0R8 Q08379 P37840 Q9C000 P18669 P00519 O00141 Q16543 P60520 Q96JB5 P02655 Q9UMX0 P40337 P21796 P10909
**5102**	receptor binding	1.48E-12	74	22.6	Q9H0E2 Q96DZ9 Q15654 O95477 P07900 P10997 P01160 P62993 P02649 Q9Y6K9 P01834 Q92731 P02647 P04040 Q30201 Q15582 P02766 P78352 P01236 P07437 P01275 P09936 Q92529 P78536 Q08431 P19338 P37231 P11142 Q00987 P42858 P01308 P00734 P54829 P02679 P05067 P42574 P08138 P0DJI8 P49407 P02751 P27797 Q99714 P01023 P52333 Q05193 Q92913 Q5MNZ9 P29353 P32121 P58753 P10451 P14136 Q9Y4E8 P60033 P01857 P09471 P03372 P02787 P01137 P01258 P60953 O00459 O75955 Q07325 Q9H0R8 P35228 Q93074 Q969D9 Q9NPJ6 P60520 P02656 Q03518 P61812 P02652
**1540**	beta-amyloid binding	4.86E-12	13	4.0	Q9Y287 P36544 P56817 O00213 Q02410 Q92870 P02649 P02647 O95704 P08138 P61812 Q99714 P01034
**32403**	protein complex binding	3.71E-10	51	15.5	P07900 Q9UL18 P10636 P35579 Q8NI77 Q02410 Q15582 P78352 P07437 P07711 P78536 P50502 Q08431 P11142 P01308 P01106 P46379 P00533 Q66K74 P02511 P42574 Q15113 P49407 P02751 P27797 P46459 Q05193 Q92913 Q13813 P29353 P32121 Q96L34 Q08345 P14136 P60033 P09471 P03372 Q8N6L0 P23142 Q08379 P37840 O00213 P55899 P00519 P08670 P60520 P07585 Q96JB5 Q8WYR1 P04156 P21796
**5488**	binding	9.03E-10	314	95.7	Q9H0E2 Q15776 Q15654 P11686 P13984 Q9NX24 P07196 Q9ULW3 Q92731 P01876 P60059 P49810 P01871 P09936 O43681 Q07343 Q08431 P68104 P42858 Q9Y2H9 Q9ULX3 P00533 O14798 Q9UHP3 Q9BSJ6 P52333 P00450 Q8N205 Q13813 Q9Y287 P62937 P32121 P56817 P62380 Q9Y6Q5 Q9H0S4 P10451 P14136 Q9H9D4 Q9UQB3 P09471 Q9HB58 P02745 P03372 P63104 P27448 Q9P2D7 P61964 P55854 Q16659 P01891 P31942 O43781 P20393 O43542 Q07325 P68133 Q9H0R8 Q9NP59 Q9C000 P21359 P19838 Q15572 Q9H0A6 Q96QH2 Q16543 P62945 P60520 P25713 Q9NPF8 Q03518 P41743 Q8WYR1 P10909 P04279 Q13951 O95477 Q8IZU0 Q9UL18 P54253 P36544 Q6MZP7 P35579 Q9H081 Q02410 P02649 Q9Y6K9 Q9BWG6 P02647 P04040 P02768 Q15582 P02766 P78352 Q13043 Q14011 Q12873 P07437 Q8N139 Q92529 P60709 Q15109 P22392 O43889 O14879 P11142 P01308 Q96LT9 P54829 P48634 P46695 P04053 Q66K74 P02753 P02511 Q15113 P0DJI8 P02751 Q99714 P46459 P02671 Q6UXB4 P49840 O75688 Q5VU43 P58753 Q96L34 P31751 Q8IUH5 Q92870 P60033 Q9BRK5 P47897 P02788 Q14151 P02787 P02786

					P04264 P60953 Q92876 Q8N6L0 Q13625 Q96GY3 Q96FW1 O75674 Q8NDX6 Q38SD2 Q86Y79 P37840 P10266 P18669 O00141 P02656 P02655 O95704 Q9UMX0 P02654 P40337 P61812 P02652 P04156 Q9BRJ6 P29016 P22061 Q96DZ9 O96006 P07900 P10997 P10636 O43399 Q6UXH1 P29372 Q9Y3Q8 Q8NI77 P62993 P01236 Q15388 P02686 Q16594 P62633 Q13642 P78536 P09417 Q13526 O95163 Q9BT40 P19338 P02679 P09543 P01106 P46379 P06396 Q9BRP8 P05067 Q99759 P42574 P02675 P49407 O60293 P01100 P49768 O75529 Q9BQN1 P08779 P29353 P13569 P30101 Q7Z569 Q9Y4E8 P04180 Q7Z333 P21860 O14960 P25788 P01137 P01258 Q9H4B4 P49639 Q15047 P06126 P49757 O00459 O95741 P23142 P07339 P55212 Q08379 O00213 P35228 Q9NRR5 Q93074 P08670 Q01850 P61457 P07585 Q96JB5 Q9BT17 P01160 P50053 Q9P013 Q8TD08 O94782 Q99689 Q08E93 Q99683 P01834 Q30201 Q96A73 P61626 P69905 Q969X6 Q9UJW9 P01034 O75509 P01275 Q8IY92 P05412 P07711 Q8NEC5 Q68CZ2 Q9P1U0 P50502 Q9UBX2 Q96IW7 P37231 P00738 Q00987 Q96EN8 Q92484 P00734 P23634 P08138 P05783 P27797 P01023 Q05193 Q92913 Q5MNZ9 O43570 Q7L5D6 Q15517 Q8WTV0 Q8TAI1 Q08345 Q9UIF8 Q9NW64 Q58DX5 Q9P2H0 Q9UQF2 P01857 P01614 P61764 Q96RG2 P61769 O14920 P07498 Q9BSE2 Q7Z5J4 O75955 Q9HCC9 P06727 P55899 Q8TAG9 P00519 Q969D9 A1E959 Q9NPJ6 P47929 Q71U36 Q15653 Q01814 Q14684 Q96NN9 P21796
**Cellular Component**					
**5615**	extracellular space	1.02E-22	87	26.3	P04279 Q96DZ9 O95477 P10997 P01160 Q6UXH1 P11686 P02649 P01834 P02647 P04040 P02768 Q30201 P01876 Q15582 P02766 P61626 P69905 P01871 P01034 P01275 P07711 P35527 P60709 Q15109 Q08431 P11142 P00738 P68104 P01308 Q92484 P00734 P02679 P09543 P00533 P06396 P02753 P05067 Q15113 P0DJI8 P02675 O60293 P02751 P0DJI9 P27797 P01023 P00450 P02671 Q9Y287 P62937 Q08345 P10451 P04180 P01857 P02747 P01614 P21860 O14960 P02745 P02788 P02787 P01137 P01258 P63104 P02786 P04264 P61769 P07498 Q92876 P23142 P07339 P06727 Q07325 Q8ND30 P68133 P37840 Q969D9 A1E959 P47929 P02656 P07585 P25713 P02655 P02654 P61812 P02652 P10909
**31982**	vesicle	1.40E-18	141	42.6	Q9H0E2 P22061 P07900 P11686 P62993 P01876 P01871 P09936 O43681 P35527 P09417 Q07343 Q08431 P19338 P68104 P42858 P02679 P09543 P00533 P06396 P05067 P02675 P49407 P49768 P00450 P08779 Q13813 Q9Y287 P62937 P32121 P13569 P56817 Q9Y6Q5 P10451 P30101 P04180 P02747 P02745 P25788 P01137

					P63104 P27448 P49757 P55854 P01891 O95741 P23142 P07339 P68133 Q9H0R8 Q08379 Q9NP59 Q9NRR5 P08670 P61457 Q16543 P60520 P25713 P41743 P10909 P04279 O95477 P50053 P35579 Q02410 P02649 P01834 P02647 P04040 P02768 Q30201 Q15582 P02766 P78352 P61626 P69905 P01034 P07437 P01275 P07711 P60709 P22392 P50502 O95084 P11142 P00738 Q00987 P01308 Q92484 P00734 P48634 P02753 P02511 P08138 P05783 Q15113 P0DJI8 P02751 P0DJI9 P27797 P46459 P01023 Q05193 P02671 Q9UKR5 Q5MNZ9 Q15517 Q8WTV0 P58753 P31751 Q08345 Q8IUH5 P60033 P01857 P01614 Q9BRK5 P61764 P02788 Q14151 P02787 P02786 P04264 P61769 P60953 O75955 Q96FW1 P06727 O75674 P37840 P18669 P47929 P02656 Q71U36 P02655 Q9UMX0 P02654 P61812 Q01814 P02652 P04156 P21796
**72562**	blood microparticle	1.40E-18	30	9.1	P00450 P02671 P02649 P01857 P02747 P01834 P01614 P02647 P02768 P01876 P02787 P01137 P63104 P02786 P04264 P69905 P01871 P60709 P06727 P68133 P11142 P00738 P00734 P02679 P06396 P02675 P02751 P02652 P10909 P01023
**31983**	vesicle lumen	1.27E-16	23	6.9	P01275 P02671 P07900 P56817 P00738 P01308 P02649 P02647 P02768 P02679 P02788 P00533 P02787 P01137 P05067 P69905 P0DJI8 P61812 P02675 P02751 P27797 P10909 P01023
**60205**	cytoplasmic membrane-bounded vesicle lumen	1.06E-15	22	6.6	P01275 P02671 P07900 P56817 P00738 P01308 P02649 P02647 P02768 P02679 P02788 P02787 P01137 P05067 P69905 P0DJI8 P61812 P02675 P02751 P27797 P10909 P01023
**43226**	organelle	4.05E-15	297	89.7	Q9H0E2 Q15776 Q15654 P11686 P13984 Q86VQ3 Q9P0N5 Q9NX24 P07196 Q9ULW3 Q92731 P01876 P60059 P49810 P01871 P09936 O43681 P35527 Q07343 Q9P2S5 Q08431 P68104 P42858 Q9Y2H9 Q9ULX3 P00533 Q9UHP3 Q9BSJ6 P52333 P00450 Q8N205 Q13813 Q9Y287 P62937 P32121 P56817 P62380 Q9Y6Q5 Q9H0S4 Q3KQU3 P10451 P14136 Q9H9D4 Q9UQB3 P02747 Q9HB58 P02745 P03372 P63104 P27448 Q9P2D7 P61964 P55854 Q16659 P01891 P31942 O43781 P20393 O43542 P68133 Q9H0R8 Q9NP59 Q9C000 P21359 P19838 Q86X19 Q15572 Q9H0A6 Q16543 P62945 P60520 P25713 Q9NPF8 Q03518 P41743 Q8WYR1 P10909 P04279 Q13951 O95477 Q8IZU0 Q9UL18 P54253 Q9NR21 Q6MZP7 P35579 Q9H081 Q02410 P02649 Q9Y6K9 Q9BWG6 P02647 P04040 P02768 Q15582 P02766 P78352 Q13043 Q14011 Q12873 P07437 Q8N139 P60709 P22392 O43889 O14879 P11142 P01308 Q96LT9 P54829 P48634 P46695 P04053 Q66K74 P02753 P02511 Q15113 P0DJI8 P02751 P0DJI9 Q99714 P46459 P02671 Q9UKR5 P49840 Q5VU43 P58753 Q96L34 P31751 Q8IUH5 Q92870 P60033 Q9BRK5 P47897 P02788 Q14151

					P02787 P02786 P04264 P60953 Q92876 Q8N6L0 Q13625 Q11203 Q96GY3 Q96FW1 O75674 Q8NDX6 Q38SD2 Q86Y79 P37840 P18669 O00141 P02656 P02655 O95704 Q9UMX0 P02654 P40337 P61812 P02652 P04156 P29016 P22061 O96006 P07900 P10636 P49662 Q6UXH1 P29372 Q9Y3Q8 Q8NI77 P62993 Q15388 P02686 Q16594 P62633 Q13642 P78536 P09417 Q9Y6X4 Q13526 O95163 Q9BT40 P19338 P02679 P09543 P01106 P46379 P06396 Q9BRP8 P05067 P42574 P02675 P49407 P01100 P49768 O75529 P08779 P29353 P13569 P30101 Q7Z569 Q9Y4E8 Q9C093 P04180 Q7Z333 P25788 P01137 P01258 Q9H4B4 P49639 Q15047 P06126 P49757 O00459 O95741 P23142 P07339 P55212 Q08379 O00213 P35228 Q9NRR5 Q93074 P08670 P61457 P07585 Q96JB5 Q9BT17 P01160 P50053 Q9P013 Q8TD08 O94782 Q99689 Q99683 P01834 Q30201 P61626 P69905 Q969X6 Q9UJW9 P01034 P01275 Q8IY92 P05412 P07711 Q8NEC5 Q9P1U0 P50502 O95084 Q9UBX2 Q96IW7 P37231 P00738 Q00987 Q92484 P00734 P23634 P08138 P05783 P27797 P01023 Q05193 Q92913 Q5MNZ9 Q15517 Q8WTV0 Q08345 Q9UIF8 Q9NW64 Q96Q45 Q9P2H0 Q9UQF2 P01857 P01614 P61764 Q96RG2 P61769 O14920 Q9BSE2 Q7Z5J4 O75955 Q9HCC9 Q15527 P06727 P00519 A1E959 Q9NPJ6 P47929 Q71U36 Q15653 Q01814 Q14684 Q96NN9 P21796
**44421**	extracellular region part	5.71E-15	135	40.8	Q9H0E2 P22061 Q96DZ9 P07900 P10997 Q6UXH1 P11686 P62993 P01876 O75829 P01871 P09936 O43681 P35527 P09417 Q08431 P19338 P68104 P02679 P09543 P00533 P06396 P05067 P02675 O60293 P00450 P08779 Q13813 Q9Y287 P62937 P13569 P10451 P30101 P04180 P02747 P21860 O14960 P02745 P25788 P01137 P01258 P63104 P27448 P55854 O95741 P23142 P07339 Q07325 Q8ND30 P68133 P08670 P61457 Q16543 P07585 P25713 P41743 P10909 P04279 O95477 P01160 P50053 P35579 P02649 P01834 P02647 P04040 P02768 Q30201 Q15582 P02766 P61626 P69905 P01034 P07437 P01275 P07711 P60709 Q15109 P22392 P50502 O95084 P11142 P00738 P01308 Q92484 P00734 P48634 P02753 P02511 P05783 Q15113 P0DJI8 P02751 P0DJI9 P27797 P46459 P01023 Q05193 P02671 Q15517 Q8WTV0 P58753 Q08345 P60033 P01857 P01614 Q9BRK5 P61764 P02788 Q14151 P02787 P02786 P04264 P61769 P07498 P60953 Q92876 O75955 Q96FW1 P06727 O75674 P37840 P18669 Q969D9 A1E959 P47929 P02656 Q71U36 P02655 P02654 P61812 Q01814 P02652 P04156 P21796

GO, Gene Ontology; AC, Accession;
***Protein Names were extracted from UniProt**
