# Supplementary material for: The amyloid interactome: Exploring protein aggregation
Source: PLoS One. 2017 Mar 1;12(3):e0173163. doi: 10.1371/journal.pone.0173163 (PMC5383009; doi:10.1371/journal.pone.0173163)
Supplement: S5 Table — The majority of proteins, which are characterised as hubs and bottlenecks are involved in signal transduction and in several metabolic processes. The most abundant GO term, though, is response to stress, in accordance with the most represented group of the amyloid interactome (See S4 Table). (PDF) [file pone.0173163.s009.pdf]

S5 Table. GO Terms enrichment of the 18 important, non-amyloidogenic hubs and bottlenecks, identified on the Amyloid Interactome.

Protein names*	Uniprot AC	GO Term				
		Molecular Chaperones	Autophagy	Metabolic Process	Response to Stress	Signal Transduction
14-3-3 protein zeta/delta	P63104				✓	✓
Beta-arrestin-1	P49407			✓	✓	✓
Epidermal growth factor receptor	P00533			✓	✓	✓
Fibronectin	P02751			✓	✓	
Gamma-aminobutyric acid receptor-associated protein-like 1	Q9H0R8		✓		✓	
Gamma-aminobutyric acid receptor-associated protein-like 2	P60520		✓		✓	
Growth factor receptor-bound protein 2	P62993			✓	✓	✓
Heat shock cognate 71 kDa protein	P11142	✓	✓	✓	✓	✓
Hsp90 co-chaperone Cdc37	Q16543	✓	✓	✓	✓	✓
Large proline-rich protein BAG6	P46379	✓		✓	✓	✓
Mitogen-activated protein kinase 6	Q16659			✓		✓
Myc proto-oncogene protein	P01106			✓	✓	✓
Myosin-9	P35579			✓	✓	✓
NF-kappa-B essential modulator	Q9Y6K9			✓	✓	✓
Peptidyl-prolyl cis-trans isomerase NIMA-interacting 1	Q13526			✓		
Serum albumin	P02768			✓	✓	✓
Transcription factor AP-1	P05412			✓	✓	✓
Vimentin	P08670					✓

GO, Gene Ontology; AC, Accession;
***Protein Names were extracted from UniProt**
