# Supplementary material for: The amyloid interactome: Exploring protein aggregation
Source: PLoS One. 2017 Mar 1;12(3):e0173163. doi: 10.1371/journal.pone.0173163 (PMC5383009; doi:10.1371/journal.pone.0173163)
Supplement: S6 Table — Proteins of the first cluster of the amyloid interactome were subjected to a GO term enrichment analysis using BiNGO [56]. The UniProtKB ACs of proteins that are characterized by overrepresented GO terms in this cluster are given in this table, along with their number and their frequency in the network. The adjusted p-value suggests the importance of these GO terms in the proteins of cluster 1. (PDF) [file pone.0173163.s010.pdf]

S6 Table. Enriched GO categories in Cluster 1 of the amyloid interactome. Enriched categories for Biological Process are those with p<10E-12, for Cellular Component with p<10E-14 and for Molecular Function with p<10E-8

GO ID	GO term	Adjusted p-value	No. of proteins	freq (%)	Proteins (Uniprot ACs)
					**Biological Process**
**6950**	response to stress	4.56E-19	91	52.0	P04279 P07900 O95477 P10636 P49662 P29372 P36544 Q86VQ3 P35579 P62993 P07196 Q99683 P02649 P01834 Q92731 P02647 P04040 P02768 P01876 P49810 P69905 P01871 Q16594 P01034 O75509 Q8IY92 P07437 P09936 P78536 P60709 P22392 O14879 P11142 P00738 Q00987 P00734 P54829 P00533 P06396 P05067 P42574 P02511 O14798 P49407 P27797 P02751 P49768 P01023 P52333 P08779 P29353 P49840 P32121 P58753 P31751 P10451 P30101 P14136 Q9UQF2 P60033 Q7Z333 P01857 P02747 P09471 P21860 P02745 P61764 P01137 P63104 O14920 P04264 O00459 P23142 O75955 O43542 Q96FW1 Q9H0R8 O00213 P37840 Q9C000 P21359 P00519 P19838 P60520 Q96JB5 P02656 Q03518 P40337 P61812 P02652 P21796
**48518**	positive regulation of biological process	8.54E-18	113	64.6	P07900 P10636 P13984 P62993 P07196 Q92731 P01876 P49810 P01871 Q16594 P78536 Q07343 P19338 P42858 P00533 P06396 P05067 Q99759 P42574 P49407 P49768 P52333 P29353 P32121 P13569 P10451 P30101 P14136 Q7Z333 P02747 P21860 P02745 P01137 P63104 P49757 P61964 O00459 P23142 O43542 P68133 Q08379 O00213 Q9NP59 Q9C000 Q93074 P21359 P19838 P08670 P61457 Q96JB5 Q03518 P41743 P04279 Q13951 O95477 P36544 P35579 Q99689 Q99683 P02649 P01834 P02647 P04040 P02768 P78352 P69905 Q9UJW9 Q13043 O75509 Q8IY92 P07711 Q68CZ2 Q9P1U0 P60709 P22392 Q9UBX2 P11142 P00738 Q00987 P00734 P54829 P27797 P02751 Q05193 P49840 P58753 Q96L34 P31751 Q8IUH5 Q9UQF2 Q92870 P60033 P01857 P01614 P61764 Q96RG2 P02787 O14920 P04264 O75955 Q13625 O75674 Q38SD2 P37840 P00519 Q9NPJ6 P02656 P02655 P02654 Q15653 P40337 P61812 P02652
**48519**	negative regulation of biological process	5.41E-16	102	58.	P10636 P62993 P07196 Q92731 P49810 O75829 P02686 Q16594 P09936 P78536 Q07343 P19338 P42858 P00533 P06396 P05067 P42574 P49407 P49768 P52333 P08779 Q13813 P32121 P10451 Q7Z569 P14136 Q7Z333 P02747 P09471 P21860 P01137 P63104 P49757 P23142 Q08379 O00213 Q9NP59 Q93074 P21359 P19838

					P08670 P60520 Q96JB5 Q03518 P41743 P04279 O95477 P54253 P36544 P35579 Q99689 P02649 P02647 P04040 P02768 P78352 Q9UJW9 Q13043 P01034 O75509 Q12873 P07437 P22392 P50502 Q9UBX2 O14879 P11142 P00738 Q00987 P00734 P54829 P05783 P02511 P27797 P02751 P01023 Q05193 Q6UXB4 P49840 O75688 P58753 P31751 Q9UQF2 Q92870 P61764 Q96RG2 O14920 P04264 Q13625 Q96FW1 O75674 Q38SD2 P37840 P00519 P02656 P02655 P02654 Q15653 P40337 P61812 P02652 P21796
**10941**	regulation of cell death	1.09E-15	56	32.0	P49662 P07196 Q99683 P02649 Q92731 P04040 P02768 P49810 P69905 Q16594 Q13043 O75509 P78536 P22392 P19338 O14879 P00738 Q00987 P42858 P54829 P00533 P06396 P42574 P05783 P02511 O14798 P49407 P27797 P02751 P49768 P52333 P49840 P32121 Q96L34 P31751 P30101 Q9UQF2 Q92870 Q7Z333 P21860 P61764 P01137 P63104 O14920 P55212 Q13625 O00213 P37840 Q9NP59 Q9C000 P21359 P00519 P19838 P40337 P61812 P41743
**43067**	regulation of programmed cell death	7.22E-15	53	30.3	P49662 P07196 Q99683 P02649 Q92731 P04040 P02768 P49810 Q16594 Q13043 O75509 P78536 P22392 P19338 O14879 Q00987 P42858 P00533 P06396 P42574 P05783 P02511 O14798 P49407 P27797 P02751 P49768 P52333 P49840 P32121 Q96L34 P31751 P30101 Q9UQF2 Q92870 Q7Z333 P21860 P61764 P01137 P63104 O14920 P55212 Q13625 O00213 P37840 Q9NP59 Q9C000 P21359 P00519 P19838 P40337 P61812 P41743
**48583**	regulation of response to stimulus	8.20E-15	91	52.0	P07900 O95477 P49662 P36544 Q99689 P62993 Q99683 P02649 P01834 Q92731 P02647 P04040 P02768 P01876 P78352 P01871 Q16594 Q13043 O75509 Q12873 P09936 P07711 P78536 P60709 Q07343 P11142 P00738 Q00987 P42858 P00734 P54829 P00533 P06396 P05067 Q99759 P42574 P02511 P49407 P27797 P02751 P49768 P01023 P52333 Q05193 Q6UXB4 P29353 P49840 O75688 P32121 P13569 P58753 Q9Y6Q5 P31751 P10451 P30101 Q7Z569 Q8IUH5 Q9UQF2 P60033 P01857 P02747 P01614 P21860 P02745 P01137 P63104 P49639 O14920 P04264 P49757 O00459 P23142 O75955 Q13625 Q96FW1 Q38SD2 O00213 P37840 Q9C000 Q93074 P21359 P00519 P19838 Q96QH2 Q96JB5 P02656 Q03518 Q15653 P61812 P02652 P21796
**42981**	regulation of apoptotic process	1.80E-14	52	29.7	P49662 P07196 Q99683 P02649 Q92731 P04040 P02768 P49810 Q16594 Q13043 O75509 P78536 P22392 P19338 O14879 Q00987 P42858 P00533 P06396 P42574 P05783 P02511 O14798 P49407 P27797 P02751 P49768 P52333 P49840 P32121 P31751 P30101 Q9UQF2 Q92870 Q7Z333 P21860 P61764 P01137 P63104 O14920 P55212 Q13625 O00213 P37840 Q9NP59 Q9C000 P21359 P00519 P19838 P40337 P61812 P41743

**19538**	protein metabolic process	4.18E-14	95	54.2	P22061 P04279 Q13951 P07900 O95477 P49662 P35579 P62993 P07196 Q99683 P02649 P01834 P02647 P04040 P02768 P01876 P49810 P78352 O75829 P01871 Q16594 Q13043 P01034 Q12873 P09936 P07711 Q92529 P78536 P22392 Q00987 Q96EN8 P68104 Q9Y2H9 P00734 P54829 P00533 P06396 P05067 Q99759 P42574 P02511 P49407 P27797 P02751 P49768 P01023 P52333 Q13813 P29353 P49840 O75688 P32121 P56817 Q96L34 P31751 P30101 Q7Z569 Q8IUH5 Q9UQF2 P04180 Q7Z333 P01857 P02747 P01614 P21860 P02745 P47897 Q96RG2 P01137 O14920 P04264 P27448 P61964 P23142 P07339 P55212 Q96FW1 O75674 Q38SD2 Q08379 O00213 P37840 Q9C000 Q93074 P21359 P00519 P19838 Q96JB5 P02656 P02655 P02654 P40337 P61812 P41743 P02652
**48523**	negative regulation of cellular process	5.93E-14	94	53.7	P04279 O95477 P54253 P35579 Q99689 P62993 P07196 P02649 Q92731 P02647 P04040 P02768 P49810 P78352 Q9UJW9 P02686 Q16594 Q13043 P01034 O75509 Q12873 P07437 P09936 P78536 P22392 P50502 Q9UBX2 P19338 O14879 P11142 P00738 Q00987 P42858 P00734 P54829 P00533 P06396 P05067 P42574 P05783 P02511 P49407 P27797 P02751 P49768 P01023 P52333 Q05193 P08779 Q6UXB4 Q13813 P49840 O75688 P32121 P31751 P10451 Q7Z569 P14136 Q9UQF2 Q92870 Q7Z333 P02747 P21860 P61764 Q96RG2 P01137 P63104 O14920 P49757 P23142 Q13625 Q96FW1 O75674 Q38SD2 Q08379 O00213 P37840 Q9NP59 Q93074 P21359 P00519 P19838 P08670 P60520 Q96JB5 P02656 P02655 P02654 Q15653 P40337 P61812 P41743 P02652 P21796
**48522**	positive regulation of cellular process	1.06E-13	99	56.6	P07900 P10636 P13984 P62993 P07196 Q92731 P01876 P49810 P01871 Q16594 P78536 P19338 P42858 P00533 P06396 P05067 Q99759 P42574 P49407 P49768 P52333 P29353 P32121 P13569 P30101 P14136 Q7Z333 P21860 P01137 P63104 P49757 P61964 O00459 P23142 O43542 Q08379 O00213 Q9NP59 Q9C000 Q93074 P21359 P19838 P61457 Q96JB5 P41743 P04279 Q13951 O95477 P36544 P35579 Q99689 Q99683 P02649 P01834 P02647 P04040 P78352 P69905 Q9UJW9 Q13043 Q8IY92 Q68CZ2 P22392 Q9UBX2 P11142 P00738 Q00987 P00734 P54829 P27797 P02751 Q05193 P49840 P58753 Q96L34 P31751 Q8IUH5 Q9UQF2 Q92870 P60033 P01857 P61764 Q96RG2 P02787 O14920 O75955 Q13625 O75674 Q38SD2 P37840 P00519 Q9NPJ6 P02656 P02655 P02654 Q15653 P40337 P61812 P02652
**50896**	response to stimulus	1.68E-13	131	74.8	P07900 P10636 P49662 P29372 P13984 Q86VQ3 P62993 P07196 Q92731 P01876 P49810 P02686 P01871 Q16594 P09936 P78536 P09417 Q96MS0 Q07343 P19338 P68104 P42858 Q9Y2H9 P00533 P06396 P05067 Q99759 P42574 O14798 P49407 P49768 P52333 P00450 P08779 Q13813 P29353 P32121 P13569 P10451 P30101 Q7Z569 P14136 P04180 Q7Z333 P02747 P09471 P21860 P02745 P01137 P63104

					P49639 P27448 Q9P2D7 O00459 P23142 O43542 P68133 Q9H0R8 O00213 Q9NP59 Q9C000 Q93074 P21359 P19838 P08670 Q96QH2 P60520 Q96JB5 Q03518 P41743 P04279 O95477 P36544 P35579 Q99689 Q99683 P02649 P01834 P02647 P04040 P02768 P78352 P69905 Q13043 P01034 O75509 Q8IY92 P07437 P07711 Q92529 P60709 P22392 O14879 P11142 P00738 Q00987 P00734 P54829 P05783 P02511 P27797 P02751 P01023 Q05193 P49840 P58753 P31751 Q8IUH5 Q9UQF2 Q92870 P60033 P01857 P01614 P61764 P02787 O14920 P04264 O75955 Q13625 Q96FW1 O75674 Q38SD2 P37840 P00519 Q9NPJ6 P02656 Q15653 P40337 P61812 P02652 P21796
**9893**	positive regulation of metabolic process	3.67E-13	76	43.4	P04279 Q13951 P07900 O95477 P36544 P13984 P35579 P62993 Q99683 P02649 Q92731 P02647 P04040 P01876 Q9UJW9 Q16594 Q13043 Q8IY92 P78536 Q9P1U0 P60709 P22392 Q9UBX2 P19338 P11142 Q00987 P00734 P54829 P00533 P06396 P05067 Q99759 P42574 P49407 P27797 P02751 P49768 P29353 P49840 P32121 P58753 P31751 P60033 Q7Z333 P21860 Q96RG2 P01137 O14920 P61964 O00459 P23142 O75955 O75674 P68133 Q38SD2 Q08379 O00213 P37840 Q9NP59 Q9C000 Q93074 P21359 P00519 P19838 P08670 Q9NPJ6 P61457 Q96JB5 P02656 P02655 P02654 Q15653 P40337 P61812 P41743 P02652
			**Molecular Function**		
**5515**	protein binding	1.03E-18	162	93.1	P22061 P07900 P10636 Q6UXH1 P29372 P13984 P62993 P07196 Q92731 P49810 P02686 P01871 Q16594 P09936 P78536 P09417 Q07343 P19338 P68104 P42858 Q9Y2H9 P00533 P06396 P05067 Q99759 P42574 O14798 P49407 O60293 P49768 P52333 P00450 Q9BQN1 P08779 Q13813 P29353 P32121 P13569 P56817 Q9Y6Q5 P10451 P30101 Q7Z569 P14136 P04180 Q7Z333 P09471 P21860 P02745 P01137 P63104 P49639 P27448 P49757 P61964 O00459 P23142 P07339 P55212 O43542 P68133 Q08379 Q9H0R8 O00213 Q9NP59 Q9C000 Q93074 P21359 P19838 P08670 Q9H0A6 Q96QH2 P61457 P60520 Q96JB5 Q03518 P41743 P04279 Q13951 O95477 P54253 P36544 P35579 Q99689 Q9H081 Q02410 Q99683 P02649 P01834 P02647 P04040 P02768 P78352 P69905 Q9UJW9 Q13043 P01034 O75509 Q12873 Q8IY92 P07437 P07711 Q92529 Q68CZ2 P60709 P22392 P50502 Q9UBX2 O14879 P11142 P00738 Q00987 Q96EN8 Q92484 P00734 P54829 P04053 P05783 P02511 P27797 P02751 Q99714 P01023 Q05193 Q6UXB4 P49840 Q15517 O75688 P58753 Q96L34 P31751 Q8IUH5 Q9UQF2 Q92870 P60033 P01857 P47897 P61764 Q96RG2 Q14151 P02787 O14920 P04264 O75955 Q13625 Q96FW1 O75674 Q38SD2 P37840 Q8TAG9 P18669 P00519 Q9NPJ6 Q71U36 P02656 P02655 O95704 Q15653 P40337 P61812

					P02652 P21796
**19899**	enzyme binding	1.17E-16	61	35.0	P07900 O95477 P10636 Q99689 P62993 P07196 Q99683 Q92731 P02647 P04040 P02768 P78352 P02686 Q16594 P01034 P07437 P09936 Q92529 P60709 P11142 Q00987 P68104 P54829 P00533 P05067 P42574 P49407 P27797 P02751 P01023 P52333 Q05193 Q9BQN1 P29353 P49840 P32121 P13569 P56817 P58753 P31751 P14136 Q9UQF2 P61764 P01137 P63104 O14920 O00459 O75955 Q96FW1 O75674 Q08379 Q9H0R8 P37840 Q9C000 P18669 P00519 P60520 Q96JB5 P02655 P40337 P21796
**42802**	identical protein binding	3.40E-16	50	28.7	P07900 P54253 P36544 P35579 P62993 P07196 Q99683 P02649 P02647 P04040 P02768 Q13043 P01034 P09417 P60709 P50502 P19338 O14879 Q00987 P42858 P00533 P05067 P02511 P02751 Q99714 Q05193 Q15517 P58753 Q7Z569 Q8IUH5 Q9UQF2 Q7Z333 P21860 P61764 Q14151 P01137 P63104 O14920 P23142 P55212 Q13625 Q38SD2 P37840 P19838 P08670 P61457 P02655 Q03518 P61812 P02652
**1540**	beta-amyloid binding	3.91E-12	11	6.3	Q02410 Q92870 P02649 P02647 P36544 P56817 O95704 O00213 P61812 P01034 Q99714
**5102**	receptor binding	2.26E-11	48	27.6	P52333 Q05193 P07900 O95477 P29353 P32121 P58753 P10451 P14136 P62993 P60033 P02649 P01857 P09471 P01834 Q92731 P02647 P04040 P01137 P78352 P02787 O00459 P07437 P09936 Q92529 P78536 O75955 Q9H0R8 P19338 Q93074 P11142 Q00987 P42858 P00734 Q9NPJ6 P54829 P60520 P02656 P05067 P42574 Q03518 P49407 P61812 P27797 P02751 P02652 Q99714 P01023
			**Cellular Component**		
**31982**	vesicle	1.68E-19	94	52.8	P22061 P04279 P07900 O95477 P35579 P62993 Q02410 P02649 P01834 P02647 P04040 P02768 P01876 P78352 P69905 P01871 P01034 P07437 P09936 P07711 P09417 P35527 P60709 P22392 P50502 Q07343 P19338 P11142 P00738 Q00987 P68104 P42858 Q92484 P00734 P00533 P06396 P05067 P05783 P02511 P49407 P27797 P02751 P49768 P01023 Q05193 P00450 P08779 Q13813 Q15517 P32121 P13569 P56817 P58753 Q9Y6Q5 P31751 P10451 P30101 Q8IUH5 P04180 P60033 P01857 P02747 P01614 P02745 P61764 Q14151 P01137 P02787 P63104 P04264 P27448 P49757 P23142 O75955 P07339 Q96FW1 O75674 P68133 Q08379 Q9H0R8 P37840 Q9NP59 P18669 P08670 P61457 P60520 Q71U36 P02656 P02655 P02654 P61812 P41743 P02652 P21796
**72562**	blood microparticle	1.24E-18	24	13.5	P00450 P60709 P68133 P11142 P00738 P02649 P00734 P01857 P02747 P01834 P01614 P02647 P02768 P01876 P06396 P01137 P02787 P63104 P69905 P04264

	P01871 P02751 P02652 P01023

GO, Gene Ontology; AC, Accession;
***Protein Names were extracted from UniProt**
