# Supplementary material for: The amyloid interactome: Exploring protein aggregation
Source: PLoS One. 2017 Mar 1;12(3):e0173163. doi: 10.1371/journal.pone.0173163 (PMC5383009; doi:10.1371/journal.pone.0173163)
Supplement: S1 File — Detailed description of the structure of the interactive Amyloid Interactome–Web application is available at http://83.212.109.111/amyloid_interactome. (PDF) [file pone.0173163.s011.pdf]

S1 File

Web application User guide

(http://83.212.109.111/amyloid_interactome)

In order to create an interactive network visualization, **Cytoscape.js** was used. Cytoscape.js, a graph theory library, allows for the display and manipulation of rich, interactive graphs such as the amyloid interactome. For more details please refer to Fig 1 and 2 of the main manuscript and S2 Fig on the Supporting Information Field.

In the main window the user can see the entire network and "interact" with it.

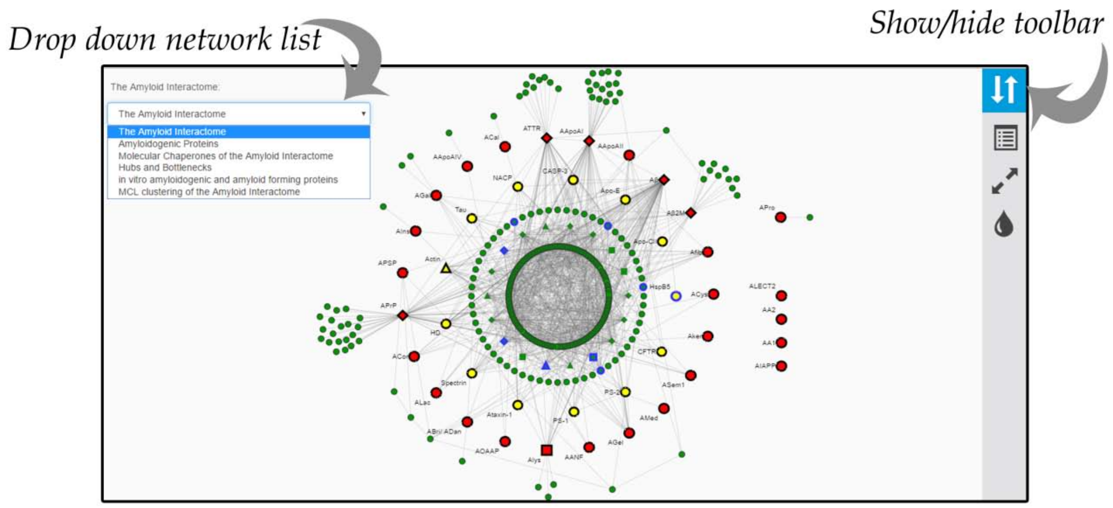

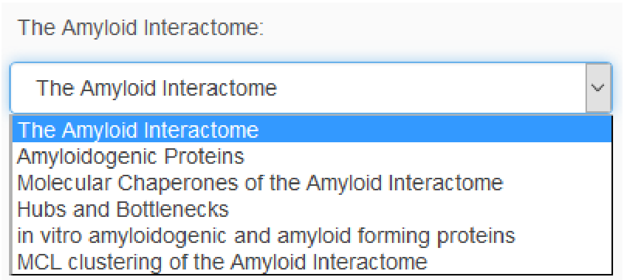

The user is allowed to select between the different networks described in the main manuscript: Fig 1 (**Amyloid Interactome**), S2 Fig (**Amyloidogenic proteins, *in vitro* amyloidogenic and amyloid forming proteins, Hubs and Bottlenecks, Molecular Chaperones of the Amyloid Interactome**) and the clustered network of Fig 2 (**MCL clustering of the Amyloid Interactome**), using the drop network list on the upper left corner of the window as shown above.

A button on the upper right corner of the screen allows the user to **show/hide the toolbar** of the application. Among the available functions, the **fit** to window button lets the user to adjust the network to its original position whereas the background colour allows the change of the network's background appearance.

Important information can be gathered from the Table Pane where the user can see all relevant information regarding selected nodes and edges, as they are deposited in the MITAB2.5 file in Intact, from where the interactions were gathered.

Specifically, the **Node Table** contains **unique identifiers** for each node (usually the UniProtKB AC), **Alternative Identifiers** for proteins (e.g. Gene Names), **Aliases** in various databases, the **NCBI Taxonomy identifier** and a column with the **Recommended UniprotKB Protein Name.** For **amyloidogenic proteins** a column with **abbreviations**, used in the main manuscript, is given, and columns indicating whether a protein is a **hub and/or bottleneck,** as well as, a column to discern between ***in vivo* and *in vitro* and amyloid forming** nodes. Part of the Node Table is shown below.

Node							
SUID	id	in_vivo_in_vitro	Hub_Bottleneck	Alias_es_interactor_A	isExcludedFromPaths	Alias_es_interactor_B	Alt_ID_s_interactor[illegible]
1637	1637	in_vivo	hub+bottleneck	psi-mi:a4_human(display_long)|Alzheimer disease amyloid protein(gene name synonym)|ABPP(gene nam[illegible]	false	psi-mi:a4_human(display_long)|Alzheimer disease amyloid protein(gene name synonym)|ABPP(gene nam[illegible]	intact:EBI-77613|Q6GS[illegible] |Q8WZ99|Q9UC33|Q9[illegible] 8|Q9UCD1|Q9UQ58|Q16[illegible] 11|Q16014|Q16019|Q[illegible]

The **Edge Table** contains **unique identifiers** for each edge, the **Interaction detection method,** the **First author** of the publication and **Identifier** of the publication in which this interaction has been detected, the **Interaction type(s),** the **Source databases** and **identifiers,** the **Interaction identifier(s)** in the corresponding source database and a **confidence score** for each interaction. Part of the Edge Table is shown below.

	Edge						
shared_interaction	Confidence_value_s_	Interaction_detection_method_s_	shared_name	Publication_1st_author_s_	Publication_Identifier_s_	Source_database_s_	name
psi-mi:"MI:0407"(direct interaction)	intact-miscore:0.41	psi-mi:"MI:0813"(proximity ligation assay)	P01236 (psi-mi:"MI:0407"(direct interaction)) P16471-7	Fleming et al. (2013)	imex:IM-20982|pubmed:23048206	psi-mi:"MI:0469"(IntAct)	P01236 (psi-mi:"MI:04[illegible]"(direct interaction)) P[illegible] 471-7
